# Arabidopsis suppressor mutant of *abh1* shows a new face of the already known players: ABH1 (CBP80) and ABI4—in response to ABA and abiotic stresses during seed germination

**DOI:** 10.1007/s11103-012-9991-1

**Published:** 2012-11-30

**Authors:** Agata Daszkowska-Golec, Weronika Wojnar, Marta Rosikiewicz, Iwona Szarejko, Miroslaw Maluszynski, Zofia Szweykowska-Kulinska, Artur Jarmolowski

**Affiliations:** 1 Department of Genetics, Faculty of Biology and Environmental Protection, University of Silesia, Jagiellonska 28, 40-032 Katowice, Poland; 2 Department of Gene Expression, Faculty of Biology, Adam Mickiewicz University, Poznan, 61-614 Poland

**Keywords:** Abiotic stresses, Abscisic acid (ABA), Arabidopsis, Seed germination, Suppressor mutant

## Abstract

Although the importance of abscisic acid (ABA) in plant development and response to abiotic and biotic stresses is well recognized, the molecular basis of the signaling pathway has not been fully elucidated. Mutants in genes related to ABA are widely used as a tool for gaining insight into the mechanisms of ABA signal transduction and ABA-dependent stress response. We used a genetic approach of a suppressor screening in order to decipher the interaction between ABH1 (CBP80) and other components of ABA signaling. *ABH1* (*CBP80*) encodes a large subunit of CBC (CAP BINDING COMPLEX) and the *abh1* mutant is drought-tolerant and hypersensitive to ABA during seed germination. The suppressor mutants of *abh1* were generated after chemical mutagenesis. The mutant named *soa1* (*suppressor of abh1 hypersensitivity to ABA 1*) displayed an ABA-insensitive phenotype during seed germination. The genetic analysis showed that the *soa1* phenotype is dominant in relation to *abh1* and segregates as a single locus. Based on *soa1*’s response to a wide spectrum of physiological assays during different stages of development, we used the candidate-genes approach in order to identify a suppressor gene. The molecular analysis revealed that mutation causing the phenotype of *soa1* occurred in the *ABI4* (*ABA insensitive 4*) gene. Analysis of pre-miR159 expression, whose processing depends on CBC, as well as targets of miR159: *MYB33* and *MYB101*, which are positive regulators of ABA signaling, revealed a possible link between CBP80 (ABH1) and ABI4 presented here.

**Electronic supplementary material:**

The online version of this article (doi:10.1007/s11103-012-9991-1) contains supplementary material, which is available to authorized users.

## Introduction

Abscisic acid (ABA) regulates a multitude of physiologically important plant responses to various stresses, as well as developmental processes throughout the plant life cycle. Among them is seed germination, a crucial phase which determines how and when plants are entered into an ecosystem and assists in their further survival (Kucera et al. 2005; Holdsworth et al. 2008). Extensive studies on ABA action during germination have uncovered major components of ABA signaling in plants (see reviews: Wasilewska et al. 2008; Umezawa et al. 2010; Daszkowska-Golec 2011). The identification of ABA receptors—PYR/PYL/RCAR (PYRABACTIN RESISTANCE 1/PYRABACTIN RESISTANCE 1-LIKE/REGULATORY COMPONENT OF ABA RECEPTOR (1) has provided a breakthrough in understanding the relations between key ABA signaling components (Ma et al. 2009; Park et al. 2009; Santiago et al. 2009; Nishimura et al. 2010). Phosphatases PP2Cs (PROTEIN PHOSPHATASES 2 C) are thought to be co-receptors of ABA (Nishimura et al. 2010; Santiago et al. 2009; Fujii et al. 2009; Melcher et al. 2009; Yin et al. 2009). However, since ABA is buried deep in the pocket of PYR/PYL/RCAR and there is no direct contact between ABA and PP2Cs, the co-receptor concept deviates from that of the classical sense in which two proteins bind the ligand (Melcher et al. 2009). PP2Cs interact with SnRK2s (SUCROSE NON FERMENTING 1 RELATED KINASES (2) and inhibit their action in the absence of ABA. SnRK2s are important for activating the transcription factors that are crucial for seed germination, such as ABI5 (ABA INSENSITIVE 5) (Fujii et al. 2007; Nakashima et al. 2009).

In *Arabidopsis thaliana* two major classes of ABA mutants have been identified: those that are insensitive and those that are hypersensitive to ABA during germination. The first group includes mutants carrying defects in gene encoding phosphatases, such as: *ABI1* (*ABA INSENSITIVE 1*) (Koornneef et al. 1984; Leung et al. 1997; Gosti et al. 1999), *ABI2* (*ABA INSENSITIVE 2*) (Koornneef et al. 1984; Leung et al. 1997), transcription factors, such as *ABI3* (*ABA INSENSITIVE 3*) (Giraudat et al. 1992; Parcy et al. 1994), *ABI4 (ABA INSENSITIVE 4)* (Finkelstein 1994; Finkelstein et al. 1998), *ABI5* (*ABA INSENSITIVE 5*) (Finkelstein 1994; Finkelstein and Lynch 2000), *CHO1* (*CHOTTO1*) (Yano et al. 2009) or components of ubiquitination machinery: *RHA2* (*RING H2*) (Bu et al. 2009), *AIRP1* (*ABA INSENSITIVE RING PROTEIN 1*) (Ryu et al. 2010). In contrast, mutations that lead to a hypersensitivity to ABA were identified in a genes including these encoding protein phosphatase 2C (AtPP2CA)—*AHG3* (*ABA HYPERSENSITIVE GERMINATION3*) (Yoshida et al. 2006), a gene encoding poly(A)-specific ribonuclease (PARN) – *AHG2* (*ABA HYPERSENSITIVE GERMINATION 2*) (Nishimura et al. 2009) and genes related to RNA metabolism, such as *SAD1* (*SUPERSENSITIVE TO ABA AND DROUGHT 1*) (Xiong et al. 2001), *HYL1* (*HYPONASTIC LEAVES 1*) (Lu and Fedoroff 2000). One of mutants related to RNA metabolism is the recessive mutant *abh1* (*ABA hypersensitive 1*), which is characterized as hypersensitive to ABA during germination and stomatal action as well as being drought tolerant. The *abh1* mutant carries the T-DNA insertion in the *CBP80* (*ABH1*) (*CAP BINDING PROTEIN 80*) gene which encodes a large subunit of the nuclear heterodimeric cap binding complex (CBC) (Hugouvieux et al. 2001; Kmieciak et al. 2002). It has been established that CBC binds to the monomethylated cap (GpppN) structure of all RNAs transcribed by RNA polymerase II and participates in the processing of polymerase II RNA primary transcripts. As recent findings have shown, CBC is not only involved in pre-mRNA splicing but also in pri-miRNA maturation (Kuhn et al. 2007; Laubinger et al. 2008; Kim et al. 2008; Szarzynska et al. 2009). It is not clear how the CBC is linked to the ABA signaling except for the known response of the identified CBC mutants (*abh1* and *cbp20*) to exogenously applied ABA. Co-immunoprecipitation experiments of Kim and co-workers (2008) revealed pri-miRNAs: 159, 166, 168 and 172 associated with CBC. It has been shown that CBP20 and CBP80 (ABH1) are necessary for ABA-dependent induction of miR159 during seed germination. Positive regulators of the ABA signaling—transcription factors MYB33 and MYB101 are downregulated by miR159. The significantly lower level of mature miR159 in *abh1* and the subsequent accumulation of *MYB33* and *MYB101* transcripts result in ABA hypersensitivity during germination (Kim et al. 2008). *MIR159* expression is regulated by ABI3, and partially by ABI5, in the presence of ABA (Reyes and Chua 2007). The action of miR159 during seed germination shows a connection between CBP80 (ABH1) and well-known ABA signaling components such as ABI3, ABI5. Despite the role of miR159 as a node linking these elements, there is still an open question as to how the CBC is involved in many aspects of the ABA signaling network.

Suppressor screens have been used successfully in Arabidopsis and other model organisms with the purpose of discovering new genes or the interactions between the ones that are already known in signaling pathways. Suppressor mutants have been used for further investigation of gene functions and for deciphering signaling pathways, including abscisic acid (Brady et al. 2003), gibberellin (Peng et al. 1999), auxin (Parry et al. 2006), biotic stress response (Kwon et al. 2004), photosynthesis (Barkan et al. 2006) or morphogenesis (Krishnakumar and Oppenheimer 1999).

In order to gain insight into the factors and pathways which interact with CBP80 (ABH1) during ABA signaling, suppressor mutants of *abh1* were generated. Suppressor mutants most likely harbor mutations in genes that function downstream or parallel to a particular gene in the studied pathway (Yoo et al. 2005). To understand the role of CBP80 (ABH1) in ABA signaling, it was hypothesized that mutations which suppress the hypersensitivity to ABA during germination may define genes whose products either interact with CBP80 (ABH1), or act in a CBP80 (ABH1)-dependent manner in this pathway. In this report, we show that inactivation of the *ABI4* (*ABA INSENSITIVE 4*) gene suppresses the *abh1* phenotype during seed germination in terms of ABA and abiotic stress response. We present a hypothetical model to decipher the connection between the ABI4 and CBP80 (ABH1) mode of action.

## Results

### Isolation of *abh1* suppressor

To gain insight into the role of CBP80 (ABH1) in ABA signaling during seed germination, genetic screening was performed in order to isolate *abh1* suppressors. The *abh1* mutant has a knockout allele with a T-DNA insertion in the *CBP80* (*ABH1*) gene resulting in a lack of transcript and consequently, of the protein of the large subunit of CBC (CAP BINDING COMPLEX) (Hugouvieux et al. 2001). The *abh1* mutant displays an ABA hypersensitive phenotype during germination in the presence of 0.4 μM ABA, a serrated leaves phenotype and drought tolerance (Hugouvieux et al. 2001, 2002). The *abh1* seeds were mutagenized with 0.25 % ethyl methanesulfonate (EMS) and M_2_ seeds insensitive to 0.4 μM ABA during germination were selected as candidates for the *abh1* suppressor. The next generation was then rescreened in order to confirm the ABA insensitive phenotype. The results of one of the selected suppressors: *soa1* (*suppressor of abh1 hypersensitivity to ABA 1*) are presented here.

### *soa1* ABA insensitivity is epistatic to *abh1* ABA hypersensitivity during germination

*soa1* (*suppressor of abh1 hypersensitivity to ABA 1*) was backcrossed to the original *abh1* mutant, and the response to ABA during germination was analyzed. The F_1_ progeny exhibited insensitivity to 0.4 μM ABA, indicating that the *abh1* suppressor mutation (*soa1*) was epistatic to *abh1*. This observation was confirmed by an analysis of F_2_ germination, which displayed a segregation ratio of 3:1 ($\chi_{3:1}^{2}$ = 0.026, *P* = 0.05) of ABA insensitive to ABA hypersensitive seedlings, respectively (Table 1). Only the suppressor gene segregated in the F_2_ progeny because both *abh1* and *soa1* carried the homozygous mutation in the *ABH1* gene.

**Table 1:** Genetic analysis of *soa1* mutant performed in the presence of 0.4 μM ABA

Generation	Number of seedlings			$\chi_{3:1}^{2}$
	Total analyzed	Insensitive to ABA (as Col-0^a^)	Hypersensitive to ABA (as *abh1*)	
F_1_ *soa1* × *abh1*	22	22	0	–
F_2_ *soa1* × *abh1*	1,218	911	307	0.026

*P* = 0.05

^a^Insensitive to 0.4 μM ABA

### *soa1* displays ABA insensitivity during seed germination and early seedling development

**Fig. 1:**
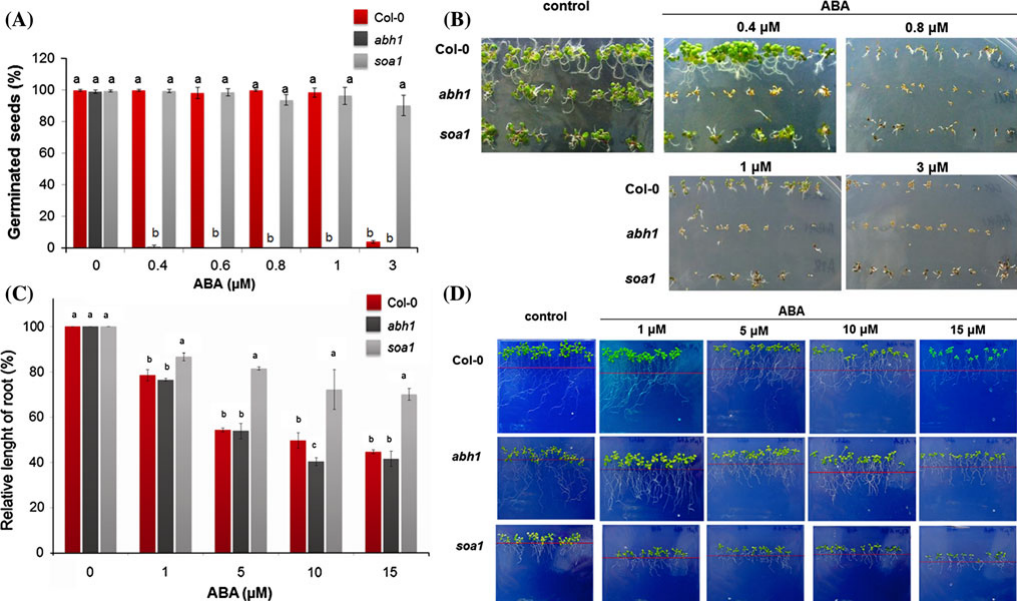
Response of Col-0, *abh1* and *soa1* plants to different ABA concentrations during seed germination and early postgerminative growth. **a**, **b** Seed germination assay on a 0.25 × MS medium containing different ABA concentrations (0.4; 0.6; 0.8; 1 and 3 μM). Values represent the mean ± SD of three biological replicates; in each 100–200 seeds of each genotype were analyzed. For each concentration means followed by the same letter do not differ significantly according to Fisher’s projected LSD (*P* = 0.05). **c**, **d** Relative root growth in the presence of different ABA concentrations. Values represent the mean ± SD of three biological replicates; in each 70–100 seedlings of each genotype were analyzed. For each concentration means followed by the same letter do not differ significantly according to Fisher’s projected LSD (*P* = 0.05). Relative root growth is expressed as the % of root growth on the control medium. The *red line* indicates the places where the root tips were after just transferring

The suppression of *abh1* hypersensitivity to ABA during germination was discovered using 0.4 μM ABA (Fig. 1a, b). In order to find out whether the response of the *soa1* suppressor to ABA can be distinguished from the reaction of the wild type Col-0, increasing concentrations of ABA (0.4; 0.6; 0.8; 1; 3 μM) were tested during germination (Fig. 1a, b). *soa1* was able to germinate and develop green cotyledons in the presence of 3 μM ABA, which completely inhibited germination of the wild-type and *abh1* (Fig. 1a, b). The ABA insensitivity of *soa1* was also observed during seedling development in relation to root elongation. In the presence of 15 μM ABA, *soa1* displayed a 25 % reduction in root length when compared to the growth on the control medium, whereas the reduction in root elongation of the wild-type and *abh1* under these conditions was 50–60 % (Fig. 1c, d).

### Salt and osmotic response of *soa1* during seed germination and early seedling development

It is known that salt and osmotic stress are able to activate similar sets of genes, and that responses to both stress factors are in some aspects ABA-dependent (Shinozaki and Yamaguchi-Shinozaki 2000; Rabbani et al. 2003).

**Fig. 2:**
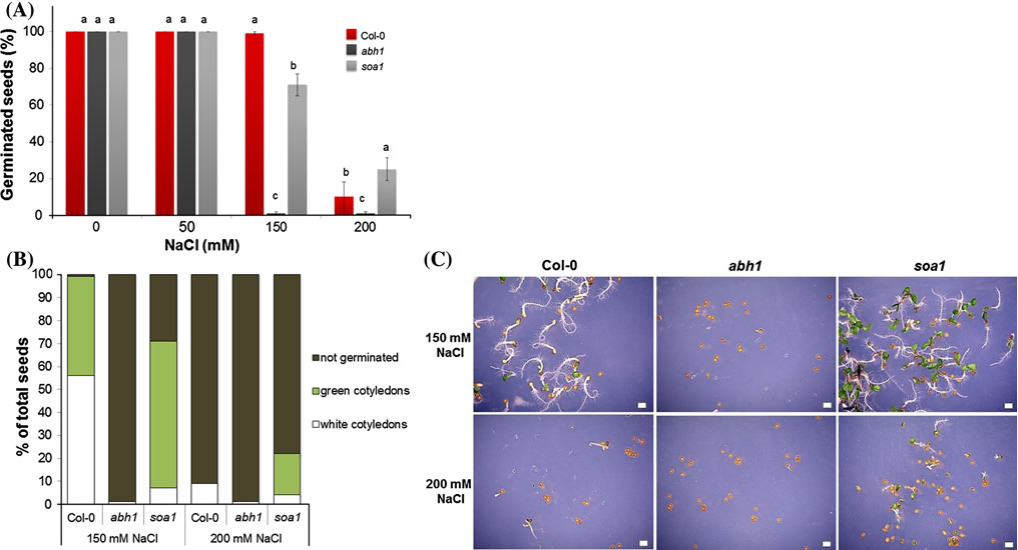
Response of Col-0, *abh1* and *soa1* plants to NaCl during seed germination and early postgerminative growth. **a** Seed germination assay on a 0.25 × MS medium containing different NaCl concentrations (50, 150 and 200 mM). **b** Cotyledon greening assay on a medium containing 150 and 200 mM NaCl. **c** Cotyledon greening assay on a medium containing 150 and 200 mM NaCl. Bleached seedlings of Col-0 and *soa1* during development in the presence of 150 and 200 mM NaCl. Values represent the mean ± SD of three biological replicates; in each 100–200 seeds of each genotype were analyzed. For each concentration means followed by the same letter do not differ significantly according to Fisher’s projected LSD (*P* = 0.05). Bar = 1 mm

To check whether there is a difference between *soa1*, *abh1* and wild-type (Col-0) in response to salt (NaCl), seeds were sown on a medium containing 50, 150 and 200 mM NaCl. A concentration of 150 mM of NaCl totally inhibited the germination of *abh1,* whereas almost all of the seeds of Col-0 and 70 % of the seeds of *soa1* germinated (Fig. 2a). Col-0, *abh1* and *soa1* were also investigated for their postgerminative development with the presence of salt in the medium. In the cotyledon-greening assay, on a medium containing 150 mM NaCl*, soa1* exhibited fully expanded green cotyledons in 90 % of the germinated seeds, while Col-0 under the same conditions displayed bleached cotyledons in 57 % of the germinated seeds (Fig. 2b, c). In the presence of higher concentration of NaCl (200 mM), *soa1* displayed fully developed green cotyledons in 81 % of the germinated seeds, while Col-0 developed only white cotyledons. This result indicated a high tolerance to salt of the *soa1* mutant (Fig. 2b, c).

**Fig. 3:**
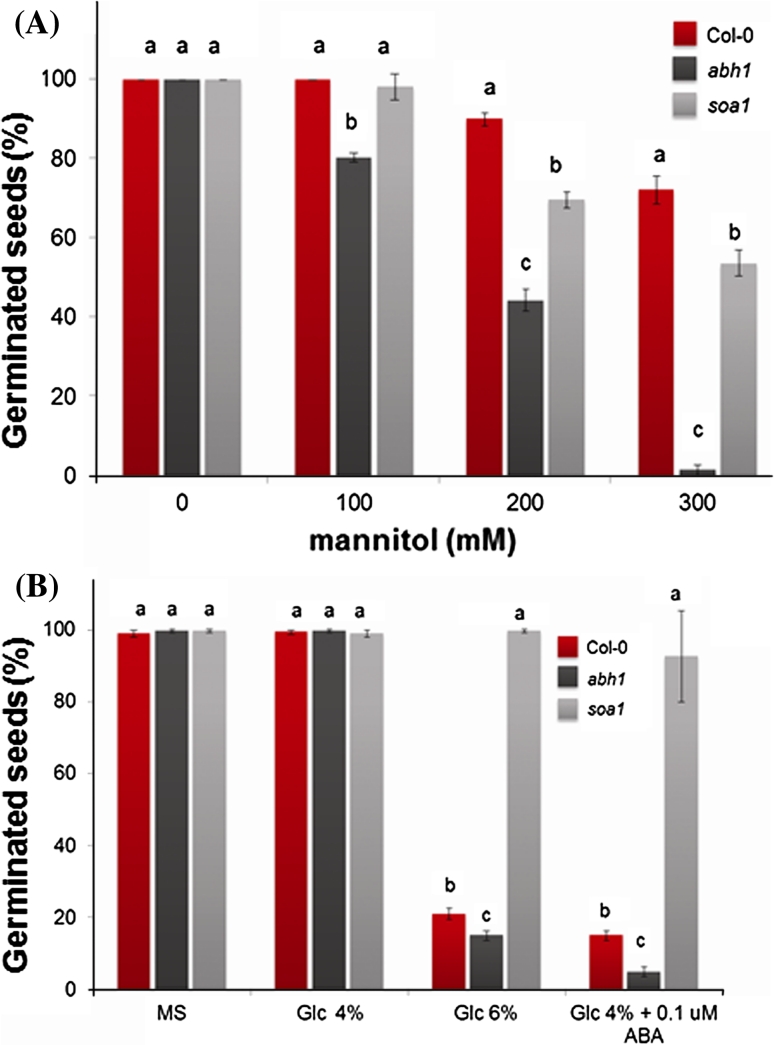
Response of Col-0, *abh1* and *soa1* plants to different mannitol and glucose concentrations during seed germination and early postgerminative growth. **a** Response of studied genotypes to different mannitol concentrations (100, 200, 300 mM) during seed germination and early postgerminative growth. **b** Response of Col-0, *abh1* and *soa1* plants to different glucose concentrations (4, 6 %, 4 % + 0.1 μM ABA) during seed germination and early postgerminative growth. Values represent the mean ± SD of three biological replicates; in each 100–200 seeds of each genotype were analyzed. For each concentration means followed by the same letter do not differ significantly according to Fisher’s projected LSD (*P* = 0.05)

In order to quantify the effect of a higher concentration of NaCl on cotyledon greening, the chlorophyll content of the seedlings was measured. This analysis confirmed the results of the germination test regarding the salt tolerance of the *soa1* mutant (Supplementary Fig. S1). To test whether *soa1* is involved in a salt-specific or general osmotic response, *abh1* and *soa1* were compared with the wild-type plant for their reaction to the osmotic effect of mannitol and glucose during seed germination and early postgerminative development. Seed germination of *soa1* showed insensitivity to the inhibitory effects of both mannitol at a concentration of 300 mM (Fig. 3a) and Glc at a concentration of 6 and 4 % with an addition of exogenous 0.1 μM ABA (the concentration noninhibitory for germination) (Fig. 3b). The addition of ABA to 4 % glucose repressed the germination of *abh1* and significantly decreased the germination of wild-type plants; however, it did not change the germination of *soa1* (Fig. 3b). A cotyledon greening assay and analysis of chlorophyll content confirmed these results (Supplementary Fig. S2). When grown on a medium containing 6 % glucose in the absence of ABA, the cotyledon greening percentages for the wild-type seedlings and *soa1* were very similar. In the presence of 0.1 μM ABA in a medium containing 4 % glucose, most of the wild-type seedlings displayed cotyledon bleaching, whereas *soa1* exhibited a slightly violet color on fully expanded green cotyledons (Supplementary Fig. S3). These results indicate that *soa1* is insensitive to high concentrations of exogenously added glucose and as a result, to *de novo* synthesized ABA, which is triggered by glucose.

**Fig. 4:**
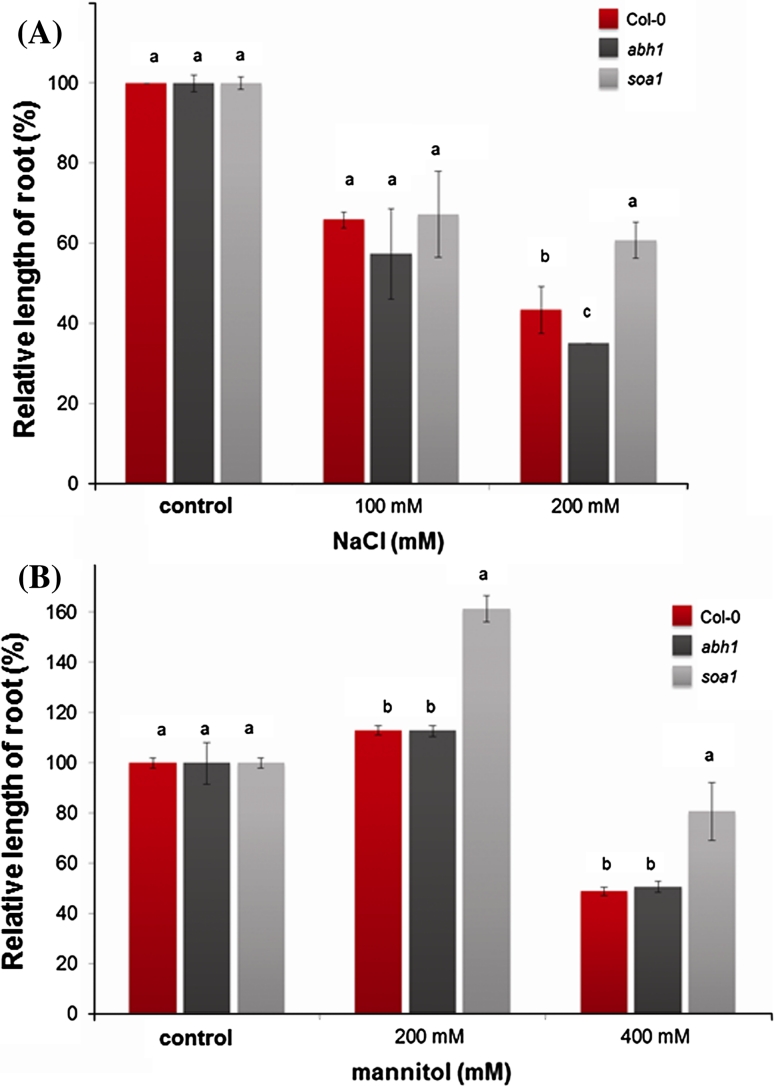
Relative root length of Col-0, *abh1* and *soa1* in the presence of salt and osmotic stress. **a** Relative root length in the presence of different salt concentrations. Values represent the mean ± SD of three biological replicates; in each 70–100 seedlings of each genotype were analyzed. For each concentration means followed by the same letter do not differ significantly according to Fisher’s projected LSD (*P* = 0.05). **b** Relative root length in the presence of different mannitol concentrations. Values represent the mean ± SD of three biological replicates; in each 70–100 seedlings of each genotype were analyzed. For each concentration means followed by the same letter do not differ significantly according to Fisher’s projected LSD (*P* = 0.05). Relative root length is expressed as the % of root length on a control medium

In addition, the *soa1* insensitivity to salt and osmotic treatment was tested during root elongation growth. *soa1* exhibited a tolerance to both salt and osmotic stress by continued root growth on a medium containing the highest used concentrations—200 mM of NaCl (Fig. 4a; Supplementary Fig. S4) and 400 mM of mannitol (Fig. 4b; Supplementary Fig. S4). It is worth noting that the parental line *abh1* is hypersensitive to most stress conditions applied during germination and early postgerminative development as compared to *soa1* (Supplementary Fig. S3).

### The effect of suppressor mutation on mature plants in drought stress

**Fig. 5:**
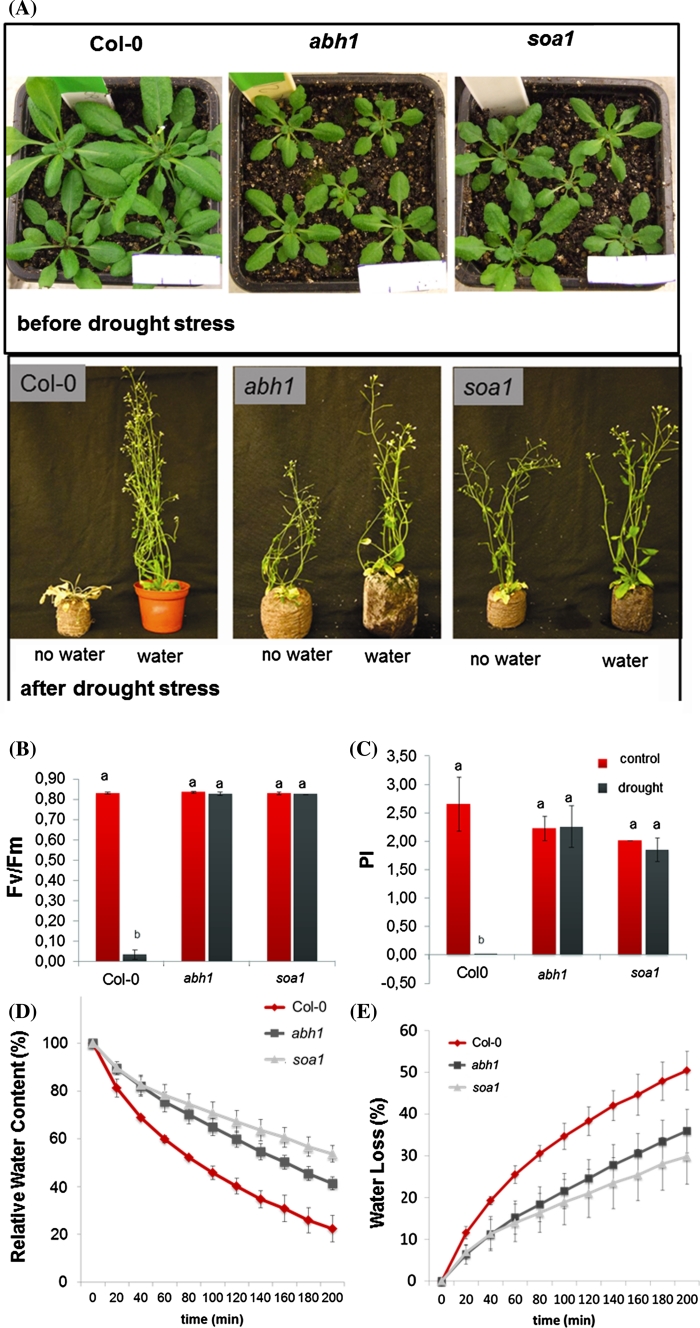
Response of Col-0, *abh1* and *soa1* mature plants to drought. **a** Representative plants of Col-0, *abh1* and *soa1*, respectively, before and after drought treatment, bar = 3 cm. **b**, **c** Fluorimetric measurements of Fv/Fm (**b**) and PI (**c**) parameters at 30th day of the assay. Values represent the mean ± SD of three biological replicates. For each genotype means followed by the same letter do not differ significantly according to Fisher’s projected LSD (*P* = 0.05). **d**, **e** Relative Water Content (**d**) and Water Loss (**e**) measured over 200 min. Values represent the mean ± SD of three biological replicates

**Fig. 6:**
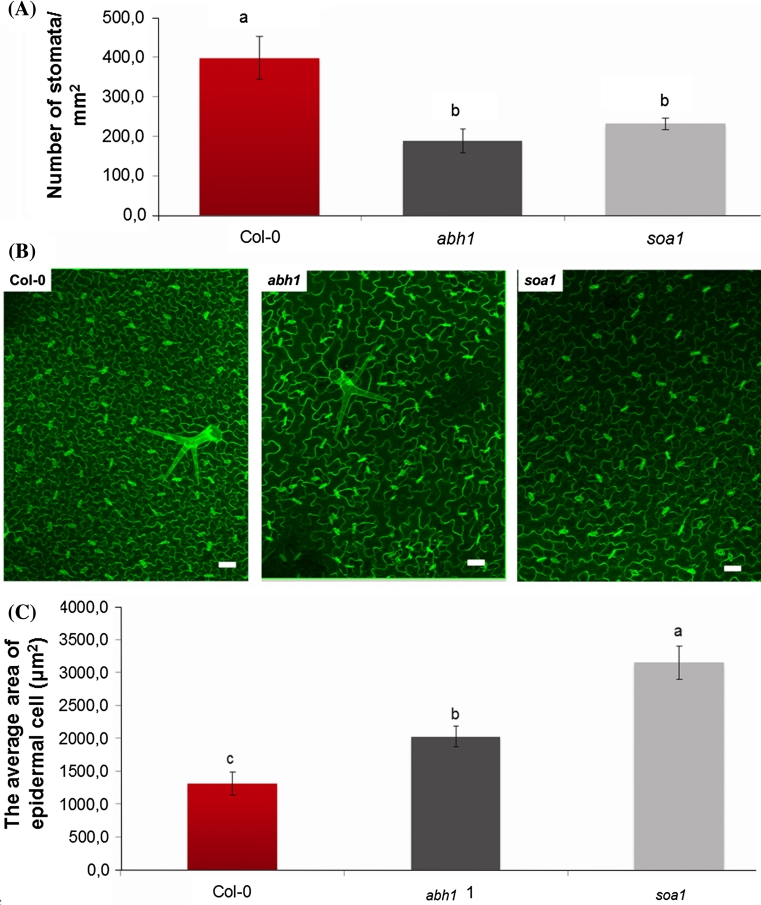
Stomatal density in rosette leaves of Col-0, *abh1* and *soa1.* **a** Stomatal density in rosette leaves. Values represent the mean ± SD of at least five leaves per experiment. Means followed by the same letter do not differ significantly according to Fisher’s projected LSD (*P* = 0.05). **b** Stomatal density in rosette leaves. Magnification ×10. Bar = 50 μm. **c** The average area of epidermal cell of Col-0, *abh1* and *soa1*. Values represent the mean ± SD of at least five leaves per experiment. Means followed by the same letter do not differ significantly according to Fisher’s projected LSD (*P* = 0.05). **d** The average length of stomata of Col-0, *abh1* and *soa1* Values represent the mean ± SD of at least five leaves per experiment. Means followed by the same letter do not differ significantly according to Fisher’s projected LSD (*P* = 0.05)

The effect of the suppression of *abh1* hypersensitivity to ABA described above was exhibited as the suppression of the *abh1* phenotype in a wide spectrum of responses to stresses applied during seed germination. To check whether the suppression also acts in other stages of plant development, mature plants of the studied genotypes were tested for their response to drought stress. The *abh1* mutant is known to be drought tolerant because of the closure of the stomata in stress conditions (Hugouvieux et al. 2001). To compare the response of Col-0, *abh1* and *soa1* to drought stress, drought treatment was applied for 4 weeks, after which the plants were re-watered for 3 days and their phenotype was observed (Fig. 5a). Col-0 did not survive the stress treatment, whereas *abh1* and *soa1* displayed a slightly wilty phenotype after 26 days of drought, but restored a normal phenotype after re-watering. Fluorimetric measurements on the 30th day of the experiment were conducted using PocketPea (Hansatech^®^). Neither the *abh1* mutant nor its suppressor mutant *soa1* displayed any drought-induced changes in PSII performance in contrast to Col-0 (Fig. 5b, c). These data are in agreement with the observed drought tolerance of *soa1*. In order to check whether the drought-tolerant phenotype of *soa1* is due to the action of the stomata, as in the *abh1*, or whether it results from the decreased stomatal density, measurements of RWC (Relative Water Content) and WL (Water Loss) in detached leaves were carried out and stomatal density was estimated using a confocal laser scanning microscope and an epifluorescent microscope. The RWC in detached leaves of both *abh1* and *soa1* was higher than in the wild-type after 200 min and WL was much slower (Fig. 5d, e). Nail polish impressions of rosette leaves examined under an epifluorescent microscope revealed that the *abh1* and *soa1* had about 50 % fewer stomata per mm^2^ than the wild-type (Fig. 6a, b). Detailed analysis using ImageJ software (ScionImage) showed that both *abh1* and *soa1* have larger epidermal cells than Col-0. The average area of an epidermal cell was 1,300 μm^2^ in Col-0 and 2,000 μm^2^ in *abh1*, whereas *soa1* displayed a 3,000 μm^2^ epidermal cell (Fig. 6c). These observations may explain the slower water loss and the higher relative water content in dehydrated leaves and, consequently, the drought-tolerant phenotype of *abh1* and its suppressor mutant *soa1.*

### Identification of *ABI4* as a suppressor gene using the candidate gene approach

The obtained results support the suggestion that the *soa1* gene encodes a protein that acts as a positive regulator of seed germination and early postgerminative development in the ABA-dependent response to stress factors. The suppressor gene seems to act mainly at the germination level because the phenotype of mature *soa1* plants treated with drought stress was very similar to the *abh1* mutant.

Based on the observed physiological reactions of the *soa1* during germination, databases were searched for genes acting in an ABA-dependent manner to salt and osmotic stresses and high levels of sugars. Six genes were selected as candidates based on their involvement in ABA signaling and mutant phenotypes insensitive to high ABA concentrations: *ABI1* (*ABA insensitive 1*), *ABI2* (*ABA insensitive 2*), *ABI3* (*ABA insensitive* 3), *ABI4* (*ABA insensitive 4*), *ABI5* (*ABA insensitive* 5) and *CHlH* (*H subunit of the Mg*-*chelatase*) (Table S1). *ABI1*, *ABI2*, *ABI3*, *ABI5* and *CHlH* genes were sequenced and then the sequences obtained were aligned between *soa1* and *abh1* using the CodonCode Aligner in order to find any mutation. None of these genes carried a point mutation in *soa1*.

However, the sequencing of the coding sequence of the *ABI4* gene revealed the presence of the point mutation C577T which caused a premature STOP codon (Supplementary Fig. S5). The identified SNP turns out to be the already known allele of *ABI4*, described as *abi4*-*101* in the Col-0 background (Laby et al. 2000). In the presented study, the same mutation induced in the *abh1* background was most likely responsible for the suppression of the *abh1* phenotype during seed germination. To check this hypothesis, it was necessary to confirm the co-segregation of the identified point mutation in the *ABI4* gene with the phenotype of the *soa1* mutant. An analysis of ABA response during germination, which was performed for 200 F_2_ plants of the *soa1* × *abh1* cross, showed that all ABA-insensitive F_2_ individuals (140) carried the identified SNP in the *ABI4*, while all ABA-hypersensitive plants (60) had a WT *ABI4* allele ($\chi_{1:2:1}^{2}$ = 5.28, *P* = 0.05). This result was confirmed by an analysis of F_3_ progeny of ABA-insensitive F_2_ plants. All F_2_ individuals which segregated for ABA-sensitivity in their progeny, carried the C577T mutation in the heterozygous state, while F_2_ plants that were homozygous for the ABA-insensitive phenotype were also homozygous for the identified mutation. Therefore, it can be concluded that the point mutation C577T in the *ABI4* gene co-segregated with the suppressor phenotype.

Additionally, genetic analysis of the cross between *abi4*-*101* and *abh1* was performed. The seeds of the F_1_ progeny of *abi4*-*101* × *abh1* cross were insensitive to 0.4 μM ABA during germination, similar to the F_1_ of *soa1* × *abh1* cross. The segregation of 15:1 of individuals insensitive to ABA (at least as Col-0) to the hypersensitive to ABA as *abh1*, observed in the F_2_ progeny of the *abi4*-*101* × *abh1* cross implies that the *abi4*-*101* allele is epistatic to *abh1* during seed germination (Table 2). Taking into account that *soa1* is insensitive to a higher ABA concentration than Col-0 (3 μM ABA), the segregation of F_2_ progeny of *abi4*-*101* × *abh1* was also tested on a medium containing 3 μM ABA. The segregation of 3:1 ABA-insensitive as *soa1 and abi4*-*101* to ABA-sensitive as Col-0 individuals confirmed the epistasis of *abi4*-*101* allele to *abh1* during seed germination in the presence of high ABA concentrations (Table 3). The performed crosses provide a definitive proof that *soa1* is a double mutant *abh1 abi4*-*101.* To maintain compatibility with the previous part of the text, we decided to keep the name *soa1* (*supressor of* *abh1 hypersensitivity to ABA 1*) for the double mutant. The name additionally indicates origin of the *soa1* through supression mutation in the *abh1* background.

**Table 2:** Genetic analysis of F_1_ and F_2_ progeny of the cross *abi4*-*101* × *abh1* performed in the presence of 0.4 μM ABA

Generation	Number of seedlings			$\chi_{15:1}^{2}$
	Total analyzed	Insensitive to ABA (as Col-0^a^)	Hypersensitive to ABA (as *abh1*)	
F_1_ *abi4*-*101* × *abh1*	56	56	0	–
F_2_ *abi4*-*101* × *abh1*	843	783	60	1.08

*P* = 0.05

^a^Insensitive to 0.4 μM ABA

**Table 3:** Genetic analysis of F_2_ progeny of the cross *abi4*-*101* × *abh1* in the presence of 3 μM ABA

Generation	Number of seedlings			$\chi_{3:1}^{2}$
	Total analyzed	Insensitive to ABA (as *soa1* ^a^)	Sensitive to ABA (as Col-0)	
F_2_ *abi4*-*101* × *abh1*	1,135	850	285	0.0073

*P* = 0.05

^a^Insensitive to 3 μM ABA

### Comparative analyses of *soa1* and *abi4*-*101* mutants response to abiotic stresses during seed germination

**Fig. 7:**
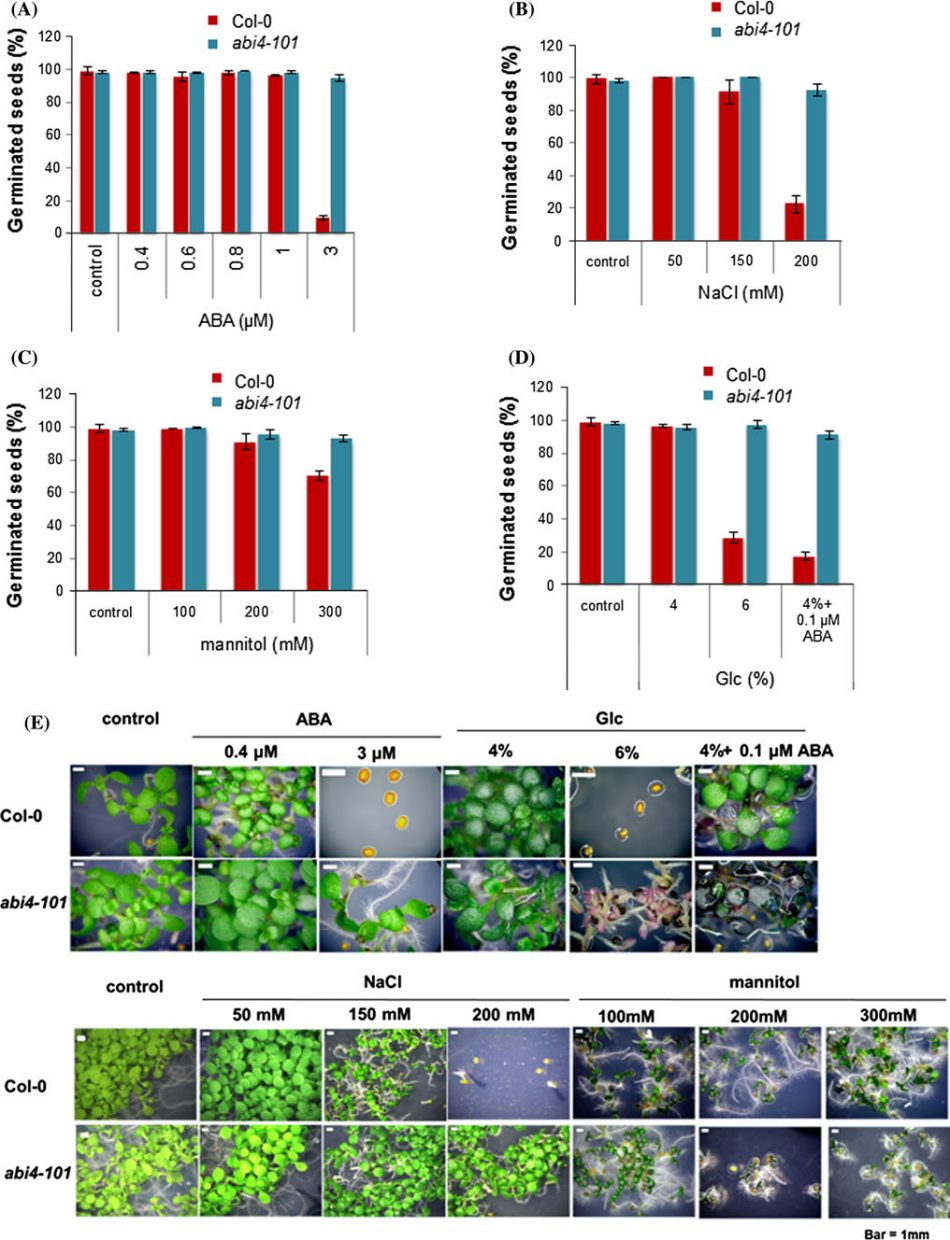
Response of the *abi4*-*101* mutant to different concentrations of ABA (**a**, **e**), NaCl (**b**, **e**), mannitol (**c**, **e**) and glucose (**d**, **e**) during seed germination and early postgerminative growth

In order to compare the response of the original *abi4*-*101* mutant with *soa1* which is a double *abh1 abi4*-*101* mutant, several physiological assays were performed during seed germination. It was shown that the *abi4*-*101* mutant exhibited a high tolerance to the doses of ABA, NaCl, mannitol and glucose applied in the study and germinated even in concentrations that drastically inhibited seed germination in the wild-type Col-0 (Fig. 7a–e). In response to ABA, glucose and glucose combined with ABA, *abi4*-*101* performed similarly to *soa1*, while seed germination in the presence of different NaCl and mannitol concentrations revealed a lower tolerance of *soa1* compared to *abi4*-*101* form.

The number of stomata in *abi4*-*101* was also investigated and turned out to be similar to the number observed in the wild-type Col-0 (Supplementary Fig. S6). Thus, it was demonstrated that the lower stomata density in the *soa1* mutant was closely associated with *abh1*, not *abi4*-*101* mutation.

### Altered expression level of ABA signaling regulators in *soa1* after ABA treatment

As the mutation in the *ABI4* gene was induced in the background of the *abh1* mutant, the obtained data shed new light on the interaction between the transcription factor ABI4 which is involved mainly in seed germination and early postgerminative development, and the CBP80 (ABH1) protein involved in RNA metabolism.

**Fig. 8:**
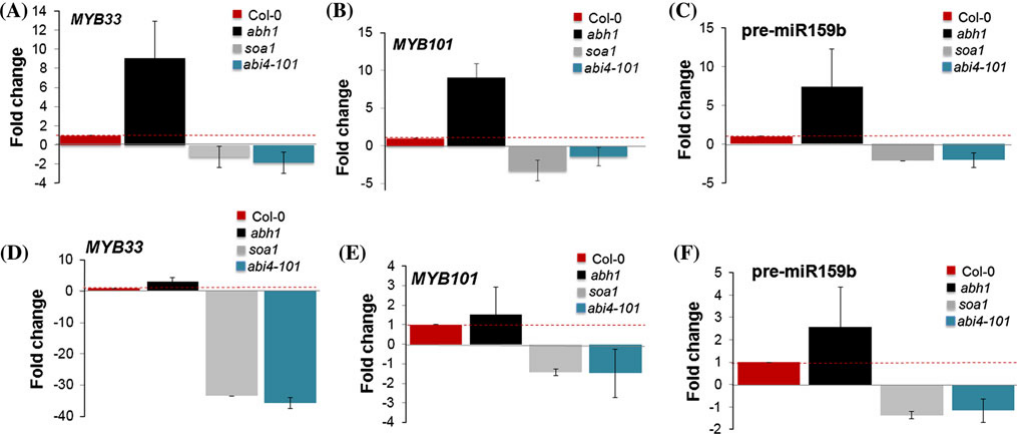
Analysis of the expression level of miR159 target transcripts—*MYB33* (**a**) and *MYB101* (**b**) and pre-miR159b (**c**) in seeds germinated in the presence of 1 μM ABA and *MYB33* (**d**), MYB101 (**e**), pre-miR159b (**f**) in 7-day-old seedlings treated with 100 μM ABA for 3 h. Data were normalized to the housekeeping gene 60S ribosomal protein L14 (*RPL14B*) (At4g27090). ΔΔCt values were then transformed out of the logarithmic scale using the formula: fold change = 2^−ΔΔCt^ (Schmittgen and Livak, 2008). Thus, mRNA values are expressed as a fold change from the wild type set to 1. The *red dotted line* shows the threshold of the wild-type expression

**Fig. 9:**
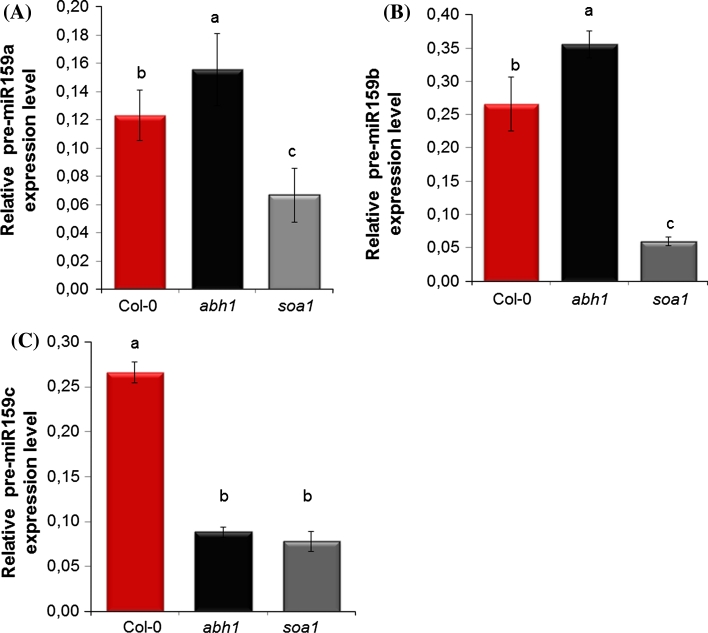
Semi-quantitative analysis of the relative transcript level of pre-miR159a, b, c in seeds germinated in the presence of 1 μM ABA. **a** Semi-quantitative analysis of the relative transcript level of pre-miR159a. **b** Semi-quantitative analysis of the relative transcript level of pre-miR159b. **c** Semi-quantitative analysis of the relative transcript level of pre-miR159c. Densitometry data for pre-miR159 mRNA were normalized to *RPL14B*. cDNA was synthesized from three independent biological replicates. The means followed by the same letter do not differ significantly according to Fisher’s projected LSD (*P* = 0.05)

To establish the interaction between ABI4 and CBP80 (ABH1), the expression of two positive regulators of ABA signaling—*MYB33* and *MYB101*—was examined in ABA-treated germinating seeds of the *soa1*, *abh1,* Col-0 and the *abi4*-*101* mutant. Additionally, the expression level of pre-miR159 (miR159 is involved in *MYB33* and *MYB101* regulation) (Allen et al. 2007; Kim et al. 2008; Reyes and Chua 2007) was determined in the same material. As expected, a Real Time-qPCR revealed a higher level of *MYB33*, *MYB101,* pre-miR159b mRNA in the *abh1* mutant compared with the wild-type. Contrary to these results, the *soa1* and *abi4*-*101* mutants exhibited a down-regulation of expression of *MYB33* and *MYB101* in the same conditions (Fig. 8a–c). The same tendency of expression was observed when young (7-day-old) seedlings were analyzed after ABA treatment (Fig. 8d–f). These data suggest that ABI4-mediated ABA signaling may be dependent on miR159 and its target genes during seed germination. The pre-miR159 is encoded by three loci, *MIR159a*, *MIR159b* and *MIR159c* but only miR159a and miR159b take part in ABA signaling during seed germination (Reyes and Chua 2007). RT-PCR performed in this study for pre-miR159a, b and c showed a higher level of pre-miR159a and pre-miR159b transcripts in the *abh1* mutant compared to the wild-type and a very low level of expression in the *soa1* (Fig. 9a–d).

**Fig. 10:**
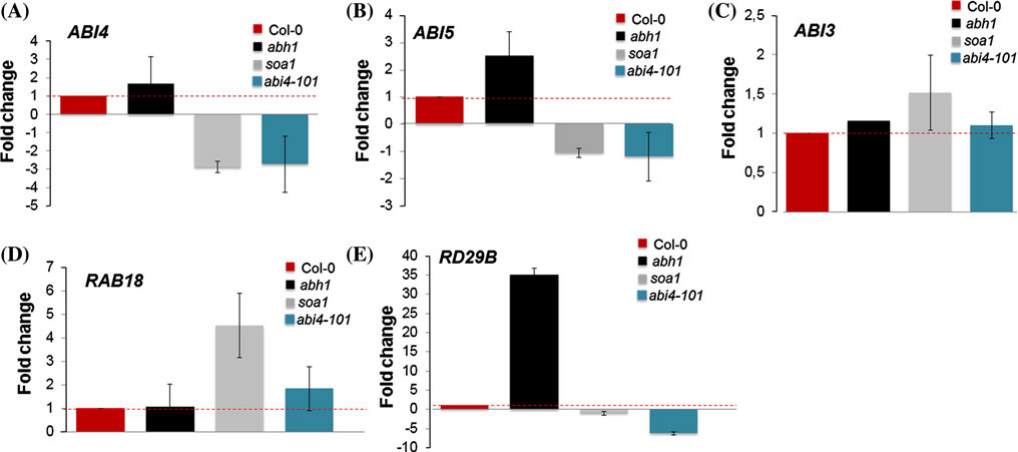
Analysis of the expression level of mRNA of *ABI4* (**a**) *ABI5* (**b**), *ABI3* (**c**), *RAB18* (**d**), *RD29B* (**e**) in seeds germinated in the presence of 1 μM ABA. Data were normalized to the housekeeping gene 60S ribosomal protein L14 (RPL14B) (At4g27090). ΔΔCt values were then transformed out of the logarithmic scale using the formula: fold change = 2^−ΔΔCt^ (Schmittgen and Livak 2008). Thus, mRNA values are expressed as a fold change from wild type set to 1. The *red dotted line* shows the threshold of the wild-type expression

**Fig. 11:**
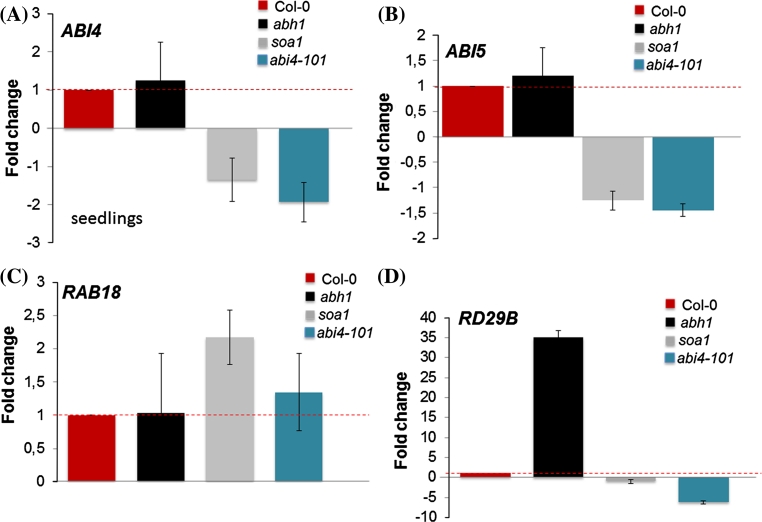
Analysis of the expression level of mRNA of *ABI4* (**a**) *ABI5* (**b**), *RAB18* (**c**), *RD29B* (**d**) in 7-day-old seedlings treated with 100 μM ABA for 3 h. Data were normalized to the housekeeping gene 60S ribosomal protein L14 (RPL14B) (At4g27090). ΔΔCt values were then transformed out of the logarithmic scale using the formula: fold change = 2^−ΔΔCt^ (Schmittgen and Livak 2008). Thus, mRNA values are expressed as a fold change from wild type set to 1. The *red dotted line* shows the threshold of the wild-type expression

ABI4 is able to activate and repress the expression of downstream genes, but also regulates its own expression. Taking into account that *soa1* carries stop mutation in the *ABI4* gene leading to a truncated protein without activation domain, the level of its expression was also evaluated. A significantly lower level of *ABI4* expression was observed in the case of both *soa1* and *abi4*-*101* when compared to Col-0 and *abh1* seeds and young seedlings developed in the presence of ABA (Figs. 10a, 11a). Summarizing, there is not enough ABI4 to activate the transcription of downstream genes and itself.

In order to investigate the expression pattern of other ABA-regulated genes, the level of expression of *ABI5*, *ABI3, RAB18* and *RD29B* were analyzed during seed germination in the presence of ABA in the Col-0, *abh1, soa1* and *abi4*-*101* forms (Fig. 10b–e). The expression of *ABI3* was not changed in *soa1* and *abi4*-*101* when compared to Col-0 and *abh1.* It should be mentioned that *ABI3* acts upstream of *ABI4*. *ABI5* and *RD29B* expression is partially regulated by ABI4 (Bossi et al. 2009; Reeves et al. 2011) and the down-regulation of these genes was observed both in *soa1* and the *abi4*-*101* mutant. The up-regulation was observed in the case of the *RAB18* gene which is one of the ABA responsive marker genes. A similar tendency of expression to that observed during seed germination was detected in 7-day-old seedlings grown on a medium containing 100 μM ABA,except for *RAB18* (Fig. 11a–d).

**Fig. 12:**
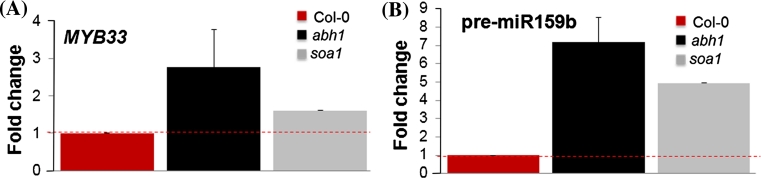
Analysis of the expression level of mRNA of *MYB33* (**a**) pre-miR159b (**b**) in 40-day-old plants treated with 30 min of rapid dehydration shock. Data were normalized to the housekeeping gene 60S ribosomal protein L14 (*RPL14B*) (At4g27090). ΔΔCt values were then transformed out of the logarithmic scale using the formula: fold change = 2^−ΔΔCt^ (Schmittgen and Livak 2008). Thus, mRNA values are expressed as a fold change from wild type set to 1. The *red dotted line* shows the threshold of the wild-type expression

Physiological analyses revealed that the *soa1* mutant is as drought tolerant as its parental line *abh1*. To provide more information about the poorly known ABI4 function in abiotic stress responses, the expression levels of the ABA-regulated gene *MYB33* and pre-miR159b were determined in response to ABA during seed germination, post-germinative growth and additionally, in mature leaves exposed to rapid dehydration stress. Both genes were down-regulated in *soa1* and *abi4*-*101* mutants in germinating seeds and young seedlings (Fig. 8a, c, d, f). However, the pattern of *MYB33* and pre-miR159b expression was completely different in mature leaves exposed to drought stress. No down-regulation was observed in *soa1* (Fig. 12a, b). These results together with the number of stomata support the hypothesis that the drought tolerance of the suppressor mutant *soa1* is not caused by the suppressor gene, *ABI4*, but is related to the action of *abh1*. Another argument supporting the conclusion that a mutation in *ABI4* gene is not able to suppress *abh1* drought tolerance comes from the in silico analysis of the *ABI4* expression pattern using public microarray data. *ABI4* expression is not induced by drought treatment and there is a lack of expression of *ABI4* in guard cells (Supplementary Fig. S7).

### *In silico* analyses of the spatial and temporal patterns of expression of *ABH1*, *ABI4*, *MYB33 and MYB101*

Altered levels of the regulators of seed germination in the presence of ABA in the *soa1* mutant led to a hypothetical model of the interaction between them and ABI4. In order to determine whether the expression of these factors is seed-specific, the online tool eFP Browser (http://bar.utoronto.ca/efp/cgi-bin/efpWeb.cgi) was used. The expression of *ABH1*, *ABI4*, *MYB33* and *MYB101* during the development of Arabidopsis was investigated. It was shown that all of the genes were expressed on a higher level in seeds (Supplementary Fig. S8A). Further analysis using the eFP Browser tool revealed that the expression of *ABH1*, *MYB33* and *ABI4* is induced in imbibed seeds (Supplementary Fig. S8B). These results supported the hypothesis about the interaction between these factors since their expression was significantly higher in seeds.

### *In silico* analyses of the promoter regions *of MIR159* and *MYB33* genes

Based on previously described results, the possible regulation of the expression of *MIR159B* or *MYB33* by ABI4 was hypothesized. An in silico analysis using PlantPAN (Chang et al. 2008) showed that the upstream region of the *MIR159B* gene contained several putative binding sites for stress-related factors, putative ABRE-like elements and a motif known as S-box. A similar analysis of the *MYB33* promoter region using Athena (http://www.bioinformatics2.wsu.edu/Athena/) revealed the presence of a putative GCC-box. These motifs are recognized as being bound by ABI4 based on literature data (Reeves et al. 2011). Although ABI4 has the ability to bind not only to sites within promoters characterized as ABI4 binding motifs, in *abi4* mutants the majority of genes with changed expression have classical ABI4-binding sites within their promoters.

## Discussion

### Mutation in *ABI4* is able to suppress the ABA hypersensitivity of the *abh1* mutant during germination

In the presented study, to analyze the role of CBP80 (ABH1) in ABA signaling during seed germination, we examined a suppressor mutant of *abh1*. It was demonstrated that the STOP mutation that occurred in glutamine 193, which leads to the truncated and nonfunctional protein of ABI4 transcription factor (Supplementary Fig. S5) crucial for seed germination in the presence of ABA, could suppress the *abh1* phenotype. Seed germination in the presence of ABA was restored in the *abh1* suppressor—*soa1* (*abh1 abi4*-*101* double mutant). It is important to mention that the suppression of *abh1* hypersensitivity to ABA is much stronger in *soa1* than in another *abh1* double mutant—*abi1*-*1 abh1* described by Hugouvieux et al. (2001). *abi1*-*1 abh1* showed only partial suppression of *abh1* hypersensitivity to ABA during germination whereas *soa1* (*abh1 abi4*-*101*) is able to germinate even in much higher concentrations of ABA than the wild-type Col-0. *soa1* mutation in the *ABI4* gene is epistatic to *abh1* hypersensitivity to ABA. The same effect was observed when the *abi4*-*101* mutant was crossed with *abh1*. The analysis of F_1_ and F_2_ progeny showed a dominant character of *abi4*-*101* to *abh1* in regards to sensitivity to ABA (Table 2, 3).

The C577T mutation in *ABI4* was identified using the candidate genes approach, in which six genes based on the *soa1* phenotype were chosen. It was demonstrated that the mutation identified in the *soa1* mutant co-segregated with its phenotype. All ABA-insensitive individuals of F_2_ progeny of the *soa1* × *abh1* cross carried the C577T mutation in the *ABI4* gene and in contrast, all hypersensitive individuals showed no *abi4* mutation. This result and the lack of insensitive plants without the *abi4* mutation or those that were hypersensitive with the *abi4* mutation strongly supports the statement that the phenotype observed in the *soa1* mutant is determined by a mutation in the suppressor gene—*ABI4*.

The identified mutation in the *ABI4* gene results in a premature stop codon and consequently ABI4 lacks a highly conserved region that includes the glutamine-rich repeat, the proline-rich repeat and the acidic domain responsible for the transcriptional activation of ABI4 (Finkelstein et al. 1998; Söderman et al. 2000). It is obvious that the mutation identified in the *soa1* mutant is crucial for the functionality of ABI4 and as a consequence, an altered gene expression regulated by ABI4 can be observed. The importance of ABI4 in seed germination under various conditions is clear from the multitude of independent reports on the identification of new *ABI4* alleles (Finkelstein 1994; Quesada et al. 2000; Huijser et al. 2000; Rook et al. 2001; Arenas-Huertero et al. 2000; Laby et al. 2000).

### The impact of mutations in *ABI4* and *ABH1* genes on the phenotype of *soa1*

The alleles of *ABI4* (Supplementary Table S2; Supplementary Fig. 9) have been identified using different physiological assays such as: response to high sugar concentrations (Huijser et al. 2000; Rook et al. 2001; Arenas-Huertero et al. 2000; Laby et al. 2000), ABA (Finkelstein 1994; Quesada et al. 2000), salt and osmotic factors (Quesada et al. 2000, 2002) during germination and early seedling development. As the aim of the presented study was to characterize the suppressor mutant *soa1* and to identify the suppressor gene, a wide spectrum of physiological assays, not only sugar, ABA or salt response, were applied in these analyses.

The sensitivity to ABA during seed germination was used as a physiological marker that enabled the isolation of the *soa1* mutant. *soa1* was not only insensitive to the concentration that inhibited the germination of *abh1*—0.4 μM, but was also able to germinate in the presence of 3 μM ABA, which inhibited Col-0 germination. An ABA sensitivity assay was used by Finkelstein (1994) and resulted in the isolation of the first *abi4* mutant—*abi4*-*1*. Response to ABA was also used by Quesada et al. (2000), although as a second criterion of selection, and resulted in the identification of *sãn5* (*abi4*-*2*). Both *abi4*-*1* and *sãn5* (*abi4*-*2*) were able to germinate in the presence of 3 μM of ABA similar to *soa1*. In an analysis of *sis5*-*1* (*abi4*-*101*), the same allele as was identified in the presented study was able to germinate in the presence of a high ABA concentration (Laby et al. 2000).

Salt, which acts in an ABA-dependent and ABA-independent manner, is known to be an inhibitor of germination and postgerminative growth (Zhu 2002). Selection in the presence of a high concentration of salt was another physiological marker that enabled the isolation of the previously mentioned *sãn5* (*abi4*-*2*) (Quesada et al. 2000). It is worth noting that *soa1* displayed a similar level of insensitivity to salt as the *sãn5* mutant. All of the identified *abi4* mutants (except for *isi3*-*1* and *isi3*-*2*) and *soa1* presented in this study were able to germinate, develop green cotyledons and continue to grow in the presence of a high concentration of glucose (Laby et al. 2000; Söderman et al. 2000; Huijser et al. 2000; Arenas-Huertero et al. 2000). The inhibitory effect of glucose in respect to germination is not caused by osmotic stress since equimolar concentrations of sorbitol or mannitol are less effective than glucose (Dekkers et al. 2004). The independence of glucose and mannitol signaling observed by Leon and Sheen (2003) was also detected in the presented study. A high concentration of mannitol, 300 mM, did not cause as dramatic an inhibition of development of the wild-type as an equimolar concentration of glucose. Glucose acts in a slightly different way than mannitol because in high concentrations it is able to induce ABA biosynthesis (Rook et al. 2001) and reduce ABA catabolism (Zhu et al. 2009). Consequently, the accumulation of ABA results in a delay of germination and an inhibition of early postgerminative development (Price et al. 2003; Dekkers et al. 2008). Cheng et al. (2002) used a selection of mutants in the presence of 4 % glucose and the same concentration of glucose but with the addition of a non-inhibitory concentration of ABA (0.1 μM). Both the *abi4* analyzed by Cheng et al. (2002) and *soa1* presented in this study were able to germinate and develop green cotyledons even when ABA was added to a medium already containing 4 % Glc. ABA and glucose insensitivity together with the salt response of *soa1* were the main reasons for selecting *ABI4* as a candidate gene for also identification of suppressor mutation.

When detailed physiological assays were performed, it was observed that *soa1* displayed shorter roots when compared to its parental line *abh1* and wild-type Col-0. A similar phenotype was also observed by Ramon et al. (2007) in the case of *abi4* mutant. Shkolnik-Inbar and Bar-Zvi (2010) showed that ABI4 is involved in ABA and cytokinin inhibition of lateral root formation. They observed increased number of lateral roots in *abi4* mutant. We did not count lateral roots in *soa1*. A common trait between *soa1* and *abh1* is the phenotype of leaves. *abh1* produces serrated leaves due to the presence of a T-DNA insert within *ABH1* gene and because *soa1* mutation was induced in an *abh1* background, *soa1* mutant also displayed serrated leaves (Supplementary Table S3). In addition, *soa1* exhibited a lack of trichomes on stems similar to the *abi4*-*101* mutant (Supplementary Fig. S10A, http://www.arabidopsis.org/servlets/TairObject?id=114498&type=polyallele). Another morphological trait that differentiates *soa1* from its parental line *abh1* is the plant height. *abh1* is smaller that the wild-type and *soa1* (Supplementary Fig. S10B).

Suppression of *abh1* by the *abi4* mutation is limited to a narrow developmental window that includes germination and early seedling development. *soa1* was found to be drought tolerant like *abh1*. Both *abh1* and *soa1* displayed the same phenotype of reduced stomata density when compared to the wild-type. It was shown that the lower number of stomata correlates with improved drought tolerance (Yang et al. 2011). We can hypothesize that the ABA hypersensitivity of *abh1* stomata together with a reduced stomata density ensures an *abh1* drought tolerant phenotype.

### ABI4 as a transcription regulator and a hypothetical model of ABI4 and CBP80 (ABH1) interaction

The response of *abi4* mutants to glucose and ABA suggests that ABI4 regulates the expression of genes involved in these pathways. ABI4 can act both as a repressor of some PhANG (Photosynthesis Associated Nuclear Genes) genes (Acevedo-Hernández et al. 2005) and as an activator of ABI5 and itself (Söderman et al. 2000; Bossi et al. 2009).

**Fig. 13:**
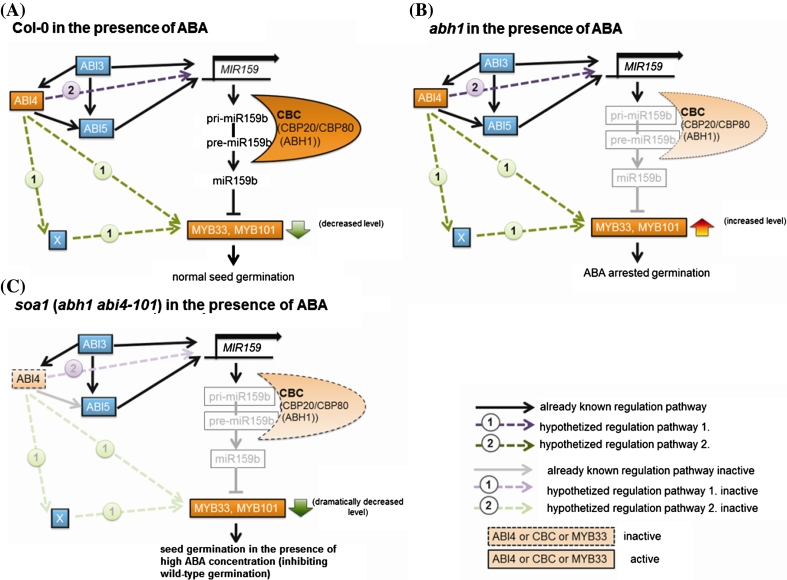
Working model of the interaction between ABI4 and CBP80 (ABH1) during seed germination. **a** Model of action in the wild type. **b** Model of action in *abh1*—parental line for suppressor *soa1.* **c** Model of action in *soa1*

In the homozygous *soa1* mutant, ABI4 is expected to be deficient in the activation domain because of a C577T mutation resulting in a premature codon stop. Inactivation of the transcription factor results in an altered expression of downstream genes. It was hypothesized by the authors that *MYB33*, *MYB101* and *MIR159* are some of these downstream genes during seed germination. Recent findings have shown that microRNA biogenesis is mediated by the nuclear CBP80/CBP20 complex and is regulated by some proteins encoded by ABA-related genes: ABI3 and partially by ABI5 after the ABA signal (Reyes and Chua 2007; Laubinger et al. 2008). The role of ABI4 in this process has not yet been determined, but it is known that the expression of ABI4 requires the ABI3 function in seeds during germination (Nakamura et al. 2001; Brady et al. 2003). It has also been proven that ABI4 binds the *ABI5* promoter upregulating its expression (Bossi et al. 2009). Based on the research presented here, the possible role of ABI4 in the ABA-dependent pathway involving miR159, MYB33, MYB101 and CBP80 (ABH1) during seed germination is proposed (Fig. 13). It assumes that ABI4 might promote the expression of an unknown factor upregulating *MYB33* and/or *MYB101* or directly upregulate the expression of these TFs. This is consistent with the in silico analysis of the upstream region of *MYB33* where putative motifs recognized by ABI4 were identified. In the case of the wild-type plants, there is an active pool of these TF transcripts available for miR159 cleavage, which consequently leads to normal seed germination and growth in the presence of a low ABA concentration (Fig. 13a). In the *abh1* mutant, the expression of MYBs is still upregulated by the ABI4, but due to a defect in the *CBP80* (*ABH1*) gene, there is not enough miR159 to inactivate the expression of MYBs. The abundance of MYBs results in ABA-hypersensitivity during germination in the *abh1* mutant (Fig. 13b). When both *ABI4* and *CBP80* (*ABH1*) are knocked-out, as in the case of the suppressor mutant *soa1*, the *abi4*-*101* allele can suppress the negative action of the *abh1* in the ABA signaling pathway and the double mutant can germinate in unfavorable conditions. In the *soa1* mutant, the down-regulation of the expression of MYBs because of *ABI4* mutation and the lack of cleavage by miR159 results in normal seed germination and seedling growth in the presence of ABA. The alternative proposed way of *ABI4* and *ABH1* interaction, parallel or equal to the one described above, assumes that *ABI4* might regulate the *MIR159b* expression either in a direct manner by interacting with ABRE or S-box elements within the *MIR159b* promoter, or indirectly by interacting with *ABI5* and/or *ABI3* (Fig. 13c). These conclusions are consistent with the lack of expression of pre-miR159b in the *soa1* and with data showed by Reyes and Chua (2007), who proved that ABI3 is necessary for miR159 accumulation, but that it requires interaction with other proteins to be tethered to ABA-responsive promoters.

Another important result that supports our hypothesis is the significantly lower level of *ABI4* expression observed in *soa1* when compared to Col-0 and *abh1* seeds. ABI4 can act as an activator and repressor of downstream gene expression but it also regulates its own expression. The *abi4*-*101* mutation identified in also *soa1* mutant leads to the premature stop codon and the protein lacks an activation domain. Together with the results of the expression of potential downstream genes: *MYB33*, *MYB101*, *MIR159*, it can be concluded that they are regulated in an ABI4-dependent manner. Therefore, a significantly lower level of expression *MYB33*, *MYB101*, pre-miR159b was observed in the *soa1* mutant.

It can be assumed that *ABI4* acts upstream or parallel to *ABH1* but in an ABA-dependent regulatory manner. Analysis of the upstream regions of *MIR159c* showed neither ABRE elements nor S-box, which suggests that ABI4 cannot be involved in its regulation. The level of expression of pre-miR159c did not differ in *abh1* compared with *soa1*. The obtained results are consistent with those observed by Laubinger et al. (2008) regarding the accumulation of pre-miR159a and b in an *abh1* mutant, not pre-miR159c.

The main objective of the presented study was to identify the suppressor mutation that leads to an ABA insensitive phenotype of the *abh1* mutant during germination. An attempt to elucidate the interaction between *CBP80* (*ABH1*) and the suppressor gene was also undertaken. The performed analyses that was focused mainly on seed germination under a range of applied abiotic stresses has led us to the identification of *ABI4* as a suppressor gene. The ability of the detected mutation in *ABI4* to suppress hypersensitivity to ABA in the *abh1* mutant clearly demonstrates a possible link between these genes and their products in the ABA pathway. Summarizing, our results shed light on the possible interactions between *ABI4* and *CBP80* (*ABH1*).

## Material and methods

### Plant material and growth conditions

The plant material used in this study included: insertional mutant *abh1* (*ABA hypersensitive* 1; accession Columbia-0; Hugouvieux et al. 2001), Col-0 used as a WT control, *soa1* (*suppressor of abh1 hypersensitivity to ABA 1*) and *abi4*-*101* mutant (Laby et al. 2000). Suppressor mutant *soa1* was obtained after the chemical mutagenesis of *abh1* with EMS. Throughout all of the experiments, the same procedure of plant cultivation was applied. First, seeds were sterilized with chlorine gas in a dessicator jar (Clough and Bent 1998). Seeds were then plated onto 0.25 × Murashige-Skoog salts supplemented with 1 % sucrose and solidified with 0.8 % agar in experiments where ABA was applied, or 0.5 × MS in experiments without ABA. After 3 days at 4 °C in the dark, plates were placed in a growth chamber at 22 °C with a 16-h-light/8-h-dark cycle, 40 μmol m^−2^ s^−1^ illumination. Four days after stratification, plantlets were transferred to Jiffy pots (Jiffy 7 Peat Pellet 42 mm, Jiffy 7C^®^) and grown in a growth chamber until maturity under the conditions described above.

### EMS mutagenesis and selection of suppressor mutants

Approximately 46,000 seeds of *abh1* were mutagenized with 0.25 % EMS. M_1_ plants were grown to maturity in 77 trays and the resulting M_2_ seeds were collected with each tray of plants providing a separate M_2_ pool. Because *abh1* is known for its hypersensitive response to 0.4 μM ABA during germination, the M_2_ population was screened for restored insensitivity to ABA. Putative suppressor mutants were identified in the first screen. Then, seeds of putative suppressors (M_3_) were sown on selection medium (0.25 × MS + 0.4 μM ABA) for confirmation of the insensitive phenotype. After analyses, 3 homozygous lines that displayed the suppression of *abh1* hypersensitivity to ABA during germination were isolated, among them the *soa1* (*suppressor of abh1* *hypersensitivity to ABA 1*) mutant.

### Genetic analysis

The *soa1* suppressor mutant was tested for the presence of T-DNA insertion within the *ABH1* gene using the BASTA resistance assay and PCR with specific primers to amplify the fragment containing the *ABH1* gene and T-DNA insertion. The *soa1* was resistant to BASTA, similar to the *abh1* mutant, whereas Col-0 did not survive the treatment. PCR amplification confirmed the presence of an insert within the *CBP80* (*ABH1*) gene in the *soa1* mutant. The analysis led to the conclusion that the suppressor mutation is extragenic to the *CBP80* (*ABH1*). The *soa1* mutant displayed serrated leaves, similar to the leaves of the parental line *abh1*. An analysis of the F_2_ generation of a cross between *abh1* and Col-0 showed that leaf serration was related to the presence of a T-DNA insert within the *CBP80* (*ABH1*) gene (Supplementary Table S3).

In order to establish the mode of the inheritance of the suppressor gene, the *soa1* mutant was crossed with its parental line—*abh1*. Seeds of the F_1_ and F_2_ generations were screened on a medium containing 0.4 μM ABA for the selection of ABA insensitive (at the level of Col-0) and ABA hypersensitive forms according to the procedure described above. The same selection was used in order to investigate the mode of inheritance of *abi4*-*101* when crossed with *abh1*. Additionally genetic analysis were performed with the use of 3 μM ABA for selection of ABA insensitive (at the level of *soa1* mutant) and ABA hypersensitive forms.

### DNA extraction

DNA was extracted from Arabidopsis rosette leaves using a modified C-TAB protocol (Doyle and Doyle 1987). Concentration and purity (A_260_/A_280_ ratio) were measured with a NanoDrop ND-1000™ spectrophotometer (ThermoScientific).

### Seed germination and cotyledon greening assays

For each comparison, seeds of all genotypes were planted in the same plate containing an MS medium (0.25 × MS salts, 1 % sucrose and 0.8 % agar) without or with different concentrations of ABA (0.4; 0.6; 0.8; 1; 3; 5 μM) or other stress factors, such as NaCl (50; 150 and 200 mM), mannitol (100; 200; 300 mM; 4; 6 %) or glucose (2; 6 and 4 % with addition of 0.1 μM ABA). For each experiment, three biological replicates were performed, each with three technical replicates. Experiments carried out at different times and on seeds of different plants of the same genotype were considered to be biological replicates. Three independent plates in each biological replicate were considered to be technical replicates. Seeds used for these experiments were harvested and stored at the same time. Plates were chilled at 4 °C in the dark for 3 days (stratified) and moved to 22 °C with a 16-h-light/8-h-dark cycle. The percentage of seed germination was scored on the 4th day after the end of stratification. Germination was defined as the visible emergence of the radicle through the seed coat. Cotyledon greening was recorded on the 7th–10th day after the end of stratification, depending on the experiment. Cotyledon greening was defined as visible expansion and turning green of the cotyledon. The analyses were performed using a Stemi 2000-C stereoscopic microscope (Zeiss) with an attached camera (Canon). In order to document the results, AxioVision LE (Carl Zeiss) software was used. The average number of seeds analyzed in one biological replicate was 100–200.

### Abiotic stresses during early seedling development and the mature plant stage

#### Root elongation assay in the presence of ABA, mannitol and NaCl

Six days after stratification, plantlets were transferred from 0.5 × MS with 1 % sucrose, 0.8 % agar with or without ABA (1; 5; 10; 15 μM), NaCl (100; 200 mM) and mannitol (200; 400 mM). Plates were incubated vertically at 22 °C with a 16-h-light/8-h-dark cycle for 7 days. Then, photographs of seedlings and roots were taken. The analyses of root length were performed using ImageJ software (ScionImage). The average number of seedlings of each genotype analyzed in one biological replicate was 70–100. Each experiment was replicated three times.

#### Drought treatment, relative water content and water loss measurements

##### Drought treatment

Drought treatment was applied to 3-week-old plants at the vegetative stage by withholding watering. The drought treatment lasted 26 days. Thereafter, plants were re-watered for 3 days and analysis was performed 24 h later. The drought assay was replicated three times (three biological replicates). Each experiment included 15 plants of each genotype. Chlorophyll fluorescence from leaf tissue was measured using a PocketPea Fluorometer (Hansatech^®^). The ratio of variable fluorescence to maximal fluorescence (Fv/Fm), representing the potential quantum yield of PSII photochemistry and PSII condition PI, were measured in dark-adapted leaf tissue. Leaves on intact plants were dark adapted at 22 °C for 20 min before each measurement. Five plants of each genotype were analyzed using PocketPea in two biological replicates.

##### Relative water content and water loss measurements

Relative Water Content (RWC,  %) was calculated as the average of measurements done every 20 min during a period of 220 min according to the formula: (FW − DW)/(TW − DW) × 100 % (modified Gonzalez and Gonzalez-Vilar, 2001). Fresh Weight (FW) was obtained by harvesting and weighing freshly detached rosette leaves every 20 min. Turgid weight (TW) was obtained by putting detached rosette leaves into an eppendorf tube with de-ionized water for 16 h at room temperature, removing excess water by wiping with absorbent paper and weighing the plant material. Dry Weight (DW) was recorded after a 24 h incubation of rosette leaves at 75 °C in a dry oven.

Water Loss (WL,  %) was expressed as the percentage of the initial fresh weight of detached rosette leaves. Detached, fully expanded leaves from 4-week-old plants were incubated under the same conditions and each sample (consisting of three individual leaves) was measured in the same way as in the RWC assay. These assays were replicated three times. Each biological replicate included 5 bulks of leaves at the same developmental stage of each genotype. Three leaves are understood to comprise a bulk.

### Observation of stomatal density and preparation of stomata impressions

Stomata impressions were made using nail polish and microscope slides (Berger and Altmann 2000). Then, observations were carried out using an epifluorescent microscope (Olympus BX-41). Photographs were taken with a digital camera (Olympus C-3040) connected to the microscope. For each genotype, 10 separate fields, 0.12 mm^2^ each, of 5 leaves were observed using a 40× magnification and 10 separate fields, 0.4259 mm^2^ each, of 5 leaves were observed using a 20× magnification. Measurements of the length of stomata and the area of epidermal cells were performed using ImageJ software (ScionImage^®^). The observations of stomata were also carried out using a confocal laser scanning microscope (Olympus FV1000, 488 nm wave length); before these observations seedlings were treated with propidium iodide (1 mg/1 mL).

### Chlorophyll content

Chlorophyll was extracted by boiling about 300 mg of fresh weight seedlings in 96 % ethanol for 10 min at 80 °C (Wintermans and de Mots 1995). NanoDrop Spectrophotometry was used in the UV/VIS mode to measure the value of absorbance at 664, 648.6 and 470 nm. Chlorophyll concentration per fresh weight was calculated as described by Lichtenthaler and Buschman (2001).

### Candidate genes approach

Based on the phenotype and physiological reactions of the *soa1* mutant to the applied stressors during germination, the candidate genes which could carry the suppressor mutation were proposed (Table S1). Six genes were chosen as candidates for sequencing and sequence analysis: *ABI1, ABI2, ABI3*, *ABI4*, *ABI5* and *CHlH*. Primers were designed with Jellyfish software. The primers are listed in Supplementary Table S4. The PCR profile was as follows: 94 °C—5 min; 94 °C—45 s, 60 °C—30 s, 72 °C—45 s (30 cycles), and 72 °C—5 min. PCR products were sequenced (Genomed, Poland) and then analyzed with a CodonCode Aligner (CodonCode Corporation, Dedham).

### Analysis of the co-segregation of a point mutation with the suppressor phenotype (Eco-TILLING)

Analysis of the co-segregation of the identified point mutation in the *ABI4* gene with the suppressor phenotype was performed in the F_2_ generation of the *soa1* × *abh1* cross. It was expected that 25 % of the F_2_ population displaying the ABA-hypersensitive phenotype would carry the wild-type alleles in the *ABI4* gene, whereas 75 % of the F_2_ plants insensitive to ABA would be homozygous or heterozygous for the identified mutation at a ratio of 1:2, respectively. First, F_2_ progeny were evaluated for sensitivity to ABA during germination. After screening, each described plantlet was transferred to a Jiffy pot (Jiffy 7 Peat Pellet 42 mm) and grown until maturity under the conditions presented earlier. DNA was extracted from F_2_ plants using the micro C-TAB method. The 525 bp *ABI4* gene fragment was sequenced in every one of the 60 F_2_ plants exhibiting ABA hypersensitivity (Genomed, Poland). In order to lower the cost of the experiment, the remaining F_2_ plants that exhibited the ABA insensitive phenotype during germination (140 individuals) were screened for the presence of point mutation within the *ABI4* gene using the Eco-TILLING strategy. This method allows the presence of a point mutation within a gene of interest to be identified when a mixture of DNA with a WT allele and a mutated allele is used as a template for gene amplification. The presence of a mutation in the PCR product can subsequently be detected through the heteroduplex formation and enzymatic cleavage of the created mismatches (Till et al. 2006; Kurowska et al. 2011). DNA from each individual F_2_ plant insensitive to ABA was mixed with the parental line *abh1* at a ratio of 1:1 and PCR with fluorescent labeled primers, forward 700 IRDye and reverse 800 IRDye were performed. PCR products were heated and cooled to form heteroduplexes and then digested using 0.1 × CJE (Celery Juice Extract), kindly provided by B. J. Till (Plant Breeding and Genetic Section Joint FAO/IAEA Division International Atomic Energy Agency). The obtained products were precipitated with 95 % ethanol with an addition of 1 % sodium acetate and then with 70 % ethanol. Polyacrylamide gel electrophoresis was performed using LICOR 4300 (LICOR^®^ Biosciences). To confirm the results of the F_2_ analysis, the F_3_ progeny of each ABA-insensitive F_2_ individual was examined for sensitivity to ABA during germination. This test allowed us to distinguish between F_2_ plants homozygous and heterozygous for this trait.

Additionally, each F_2_ ABA-insensitive plant was examined for the state of mutation in the *ABI4* gene: homozygous or heterozygous form. It was expected that after heating and cooling, the heterozygous F_2_ plants would create heteroduplexes in the PCR product of the amplified *ABI4* gene, whereas the homozygous ones would not.

### RNA extraction and gene expression analysis

Seeds of Col-0, *abh1* and *soa1* were stratified for 2 days at 4 °C and then germinated on an MS medium with 1 μM ABA for 2 days. The analysis of gene expression in seedlings was performed using 7-day-old seedlings which were first incubated for 3 h in 100 μM ABA. In the case of mature plants, 40-day-old rosette leaves were treated with rapid dehydration in a laminar airflow for 30 min. Harvested samples were immediately frozen in liquid nitrogen and stored at −80 °C. Three biological experiments were carried out. RNA was extracted using a modified TRizol method. DNA was removed during a 30-min DNase (Promega) treatment. For gene expression analysis cDNA was made using a RevertAid™ First Strand cDNA Synthesis Kit (Fermentas). qPCR was performed with a LightCycler Fast Start DNA Master SYBR Green I kit (Roche) using a Roche Light Cycler Real Time PCR machine. Standard curves were generated for each gene of interest. Fold changes in gene expression were calculated using the delta–delta Ct method (Schmittgen and Livak 2008). Data were normalized to the housekeeping gene 60S ribosomal protein L14 (RPL14B) (At4g27090) (Yamagishi et al. 2005; Walley et al. 2007). ΔΔCt values were then transformed out of the logarithmic scale using the formula: fold change = 2^−ΔΔCt^ (Schmittgen and Livak 2008). Thus, mRNA values are expressed as a fold change from wild-type set to 1. The mean value was of three independent biological replicates (each in two technical replicates). *RPL14B* expression was not different from the wild-type at any of the analyzed treatments/time-points. Gene-specific primers for detecting transcripts of *RPL14B*, *MYB3*3, *MYB101*, *ABI4, ABI3, ABI5, RD29B, RAB18* and pre-miR159a, b and c are listed in Supplementary Table S4.

For the analysis of differential expression during seed germination of pre-miR159a, b and c, RT-PCR was conducted in three biological replicates. Twenty microliters from each PCR reaction were fractionated by 2 % agarose gel in a Tris–acetate EDTA buffer and stained with ethidium bromide. The ethidium bromide stained gels were digitally photographed. The ImageJ for Windows (http://rsb.info.nih.gov/ij/) program was used to quantify the intensity of the ethidium bromide stained DNA bands from the negative images of the gels.

## Electronic supplementary material

Below is the link to the electronic supplementary material.

Supplementary material 1 (DOC 76 kb)

Supplementary material 2 (DOC 79 kb)

Supplementary material 3 (DOC 640 kb)

Supplementary material 4 (DOC 4354 kb)

Supplementary material 5 (DOC 39 kb)

Supplementary material 6 (DOC 1367 kb)

Supplementary material 7 (DOC 247 kb)

Supplementary material 8 (DOC 1535 kb)

Supplementary material 9 (DOC 108 kb)

Supplementary material 10 (DOC 2678 kb)

Supplementary material 11 (DOC 37 kb)

Supplementary material 12 (DOC 40 kb)

Supplementary material 13 (DOC 28 kb)

Supplementary material 14 (DOC 51 kb)
