# Supplementary material for: Arabidopsis suppressor mutant of abh1 shows a new face of the already known players: ABH1 (CBP80) and ABI4—in response to ABA and abiotic stresses during seed germination
Source: Plant Mol Biol. 2012 Nov 30;81(1):189–209. doi: 10.1007/s11103-012-9991-1 (PMC3527740; doi:10.1007/s11103-012-9991-1)

Figure S2. Response of Col-0, *abh1* and *soa1* plants to Glc during seed germination and early postgerminative growth. Quantification of chlorophyll content. Values represent the mean ± SD of three biological replicates, in each 100-200 seeds of each genotype were analyzed. For each concentration an asterisk means significant statistical differences according to the Student t-test (P = 0.05).


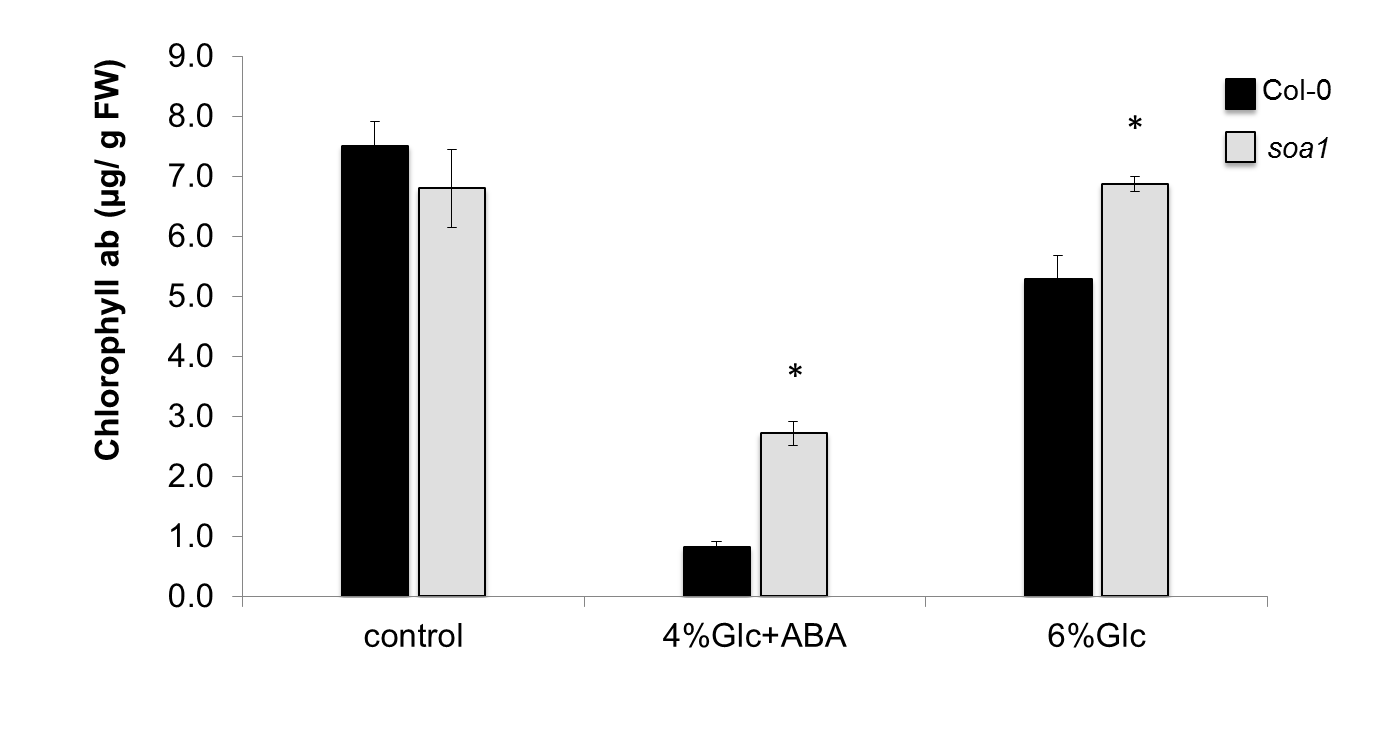

Supplement: Supplementary file 2 — Supplementary material 2 (DOC 79 kb) [file 11103_2012_9991_MOESM2_ESM.doc]
