# Supplementary material for: Arabidopsis suppressor mutant of abh1 shows a new face of the already known players: ABH1 (CBP80) and ABI4—in response to ABA and abiotic stresses during seed germination
Source: Plant Mol Biol. 2012 Nov 30;81(1):189–209. doi: 10.1007/s11103-012-9991-1 (PMC3527740; doi:10.1007/s11103-012-9991-1)

Figure S3. Response of Col-0, *abh1*, and *soa1* to glucose and mannitol during seed germination and early postgerminative growth. Photographs were taken when plants were 13 days old. Bar = 1 mm.


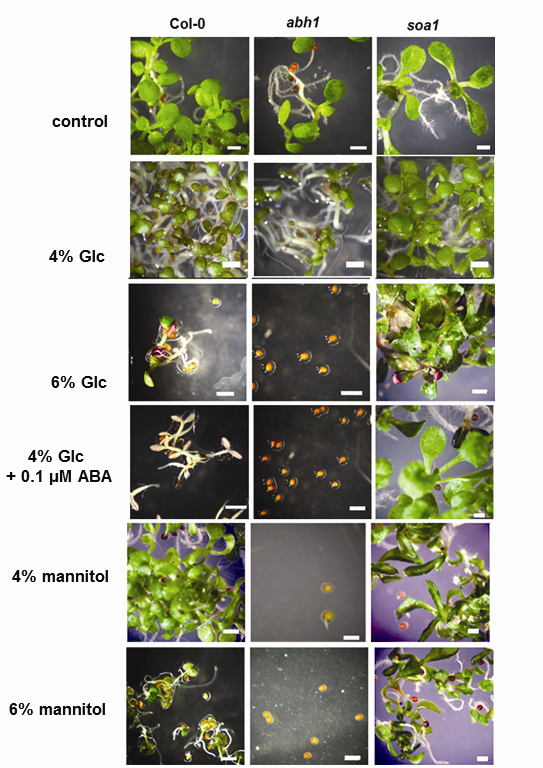

Supplement: Supplementary file 3 — Supplementary material 3 (DOC 640 kb) [file 11103_2012_9991_MOESM3_ESM.doc]
