# Supplementary material for: Arabidopsis suppressor mutant of abh1 shows a new face of the already known players: ABH1 (CBP80) and ABI4—in response to ABA and abiotic stresses during seed germination
Source: Plant Mol Biol. 2012 Nov 30;81(1):189–209. doi: 10.1007/s11103-012-9991-1 (PMC3527740; doi:10.1007/s11103-012-9991-1)

Figure S4. Relative length of root of Col-0, *abh1* and *soa1* in the presence of NaCl (**A**) and mannitol (**B**). Red line shows the beginning of the root’s growth after the transfer from control medium to NaCl or mannitol containing. Bar = 0.5 cm

**A**.


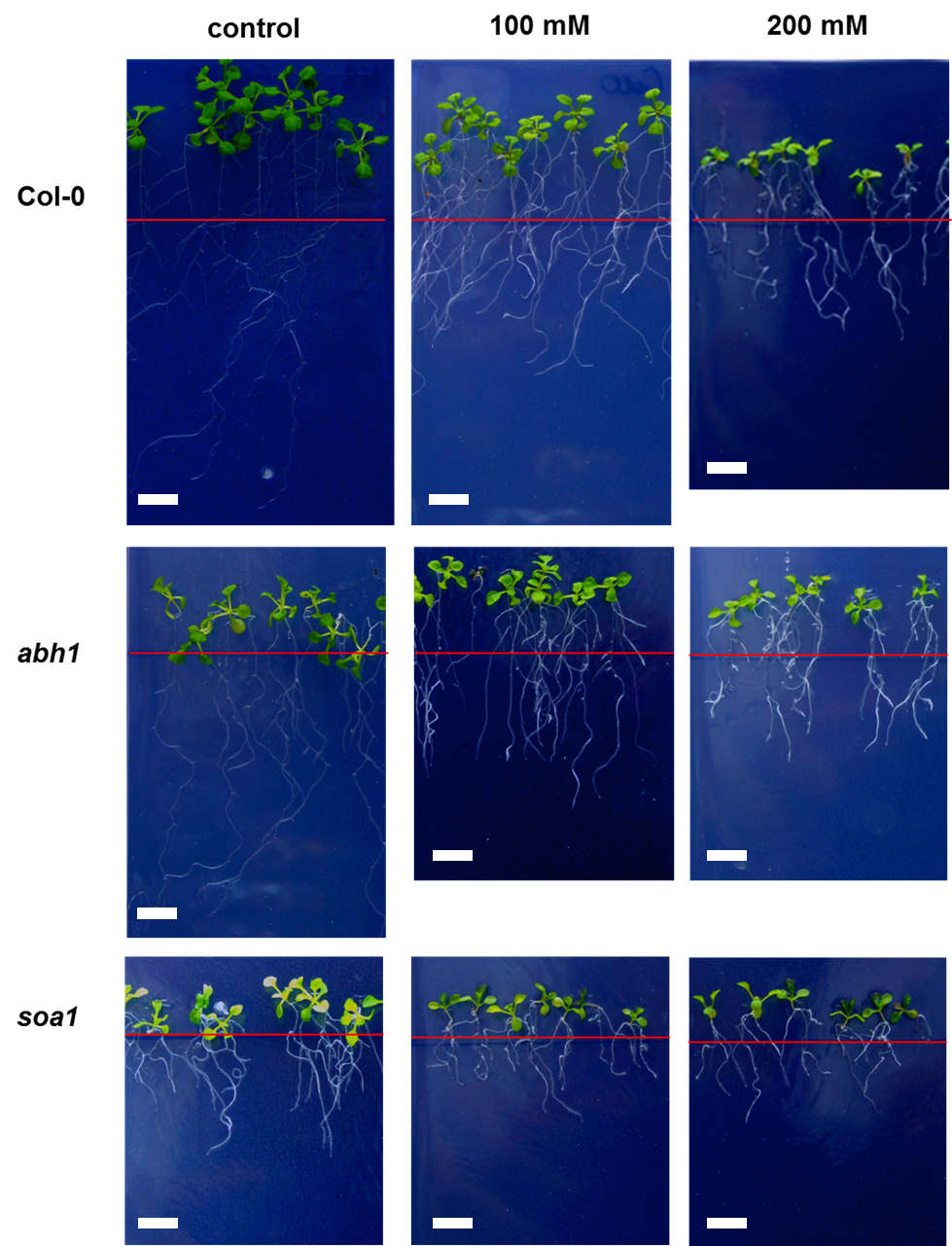


**B.**


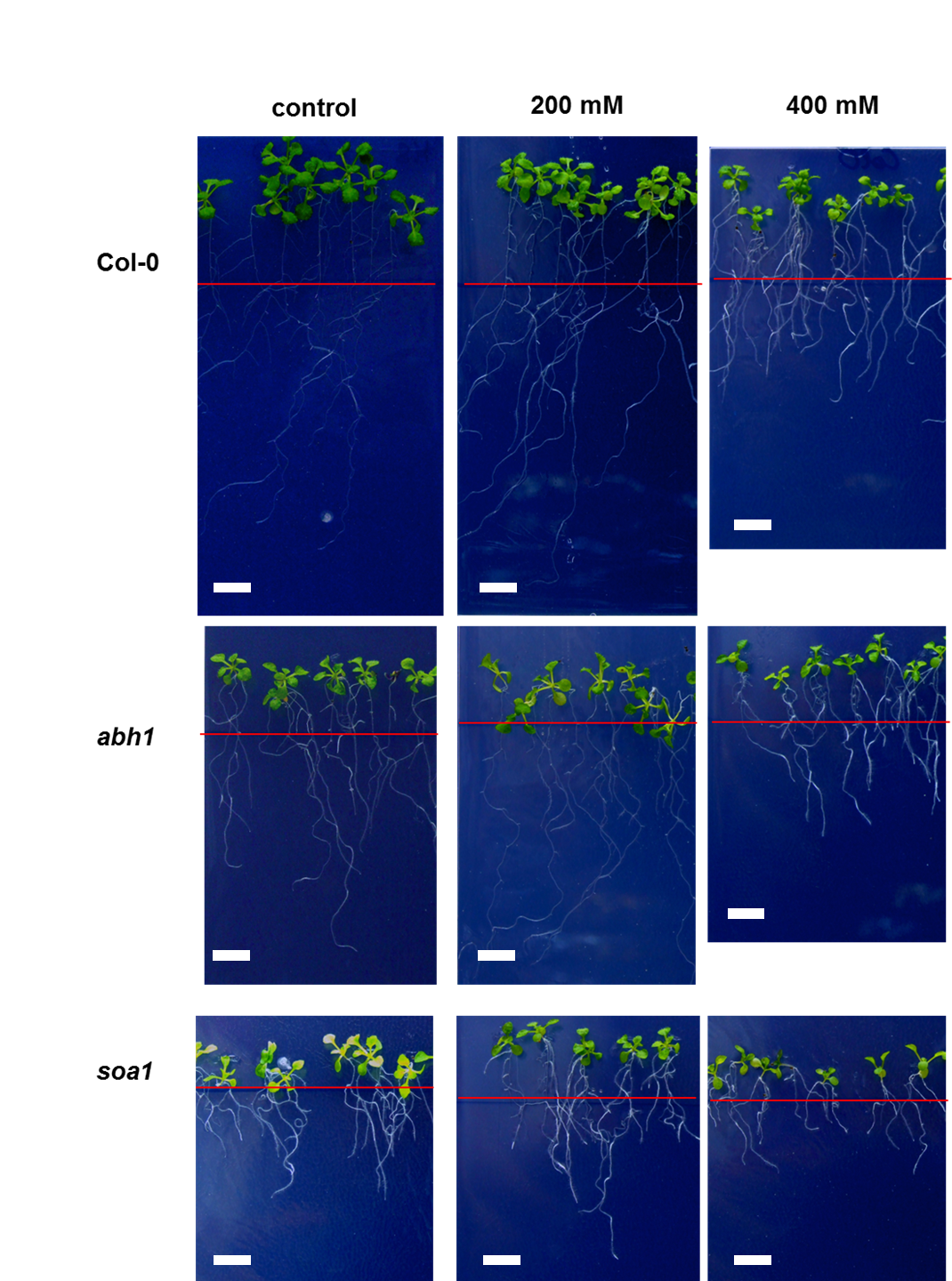

Supplement: Supplementary file 4 — Supplementary material 4 (DOC 4354 kb) [file 11103_2012_9991_MOESM4_ESM.doc]
