# Supplementary material for: Arabidopsis suppressor mutant of abh1 shows a new face of the already known players: ABH1 (CBP80) and ABI4—in response to ABA and abiotic stresses during seed germination
Source: Plant Mol Biol. 2012 Nov 30;81(1):189–209. doi: 10.1007/s11103-012-9991-1 (PMC3527740; doi:10.1007/s11103-012-9991-1)
Supplement: Supplementary file 5 — Supplementary material 5 (DOC 39 kb) [file 11103_2012_9991_MOESM5_ESM.doc]

Figure S5. Identified mutation in *soa1* mutant. ABI4 proteins alignment using MAFFT Multiple Sequence Aligned and visualised with the use of BoxShade 3.21. (At – *Arabidopsis thaliana*, Pt – *Populus trichocarpus*, Gm – *Glycine max*, Zm – *Zea mays*, Sb – *Sorghum bicolor*, AP2 – APETALA2, ERF – ETHYLENE RESPONSIVE FACTOR) with a marked fragment of protein that is affected and truncated in a *soa1* mutant. Within APETALA2 domain it was marked secondary structures such as: β-sheets (red arrows), α-helics (green wave) and loops (purle squares). It was evaluated with the use of PSIPRED (<http://bioinf.cs.ucl.ac.uk/psipred/>).

**AtABI4**/1-328 1 ------------------------------------------------------------------------------M-
**AtABI4 (soa1)** 1 ------------------------------------------------------------------------------M-
PtABI4/1-334 1 ------------------------------------------------------------------------------MD
GmSGR/1-338 1 ------------------------------------------------------------------------------M-
ZMAP2/1-248 1 ------------------------------------------------------------------------------M-
SbAP2/1-249 1 ------------------------------------------------------------------------------M-
consensus 1 ................................................................................

**AtABI4**/1-328 2 -DPLASQHQHNHL-----------EDNNQTLT--HNNPQSDSTTDSSTSSAQRKRKGKGGPDNSKFRYRGVRQRSWGKWV
**AtABI4 (soa1)** 2 -DPLASQHQHNHL-----------EDNNQTLT--HNNPQSDSTTDSSTSSAQRKRKGKGGPDNSKFRYRGVRQRSWGKWV
PtABI4/1-334 3 NSSLSHPPQEPTTTTTKLSSNEKSTDNNTTATTPTTATTSDTNSNNNSSGNSRKCKGKGGPDNGKFRYRGVRQRSWGKWV
GmSGR/1-338 2 -ASLLPQPQETKP-TATTATET--TPSETSIT--ASANKSSSSNNNNSSSNSRKCEGKGGPDNNKFRYRGVRQRSWGKWV
ZMAP2/1-248 2 ------------------------EASNNESA--PTA-EAAAGSGPAGGEGRKGKAPKGGPENGKFRYRGVRQRSWGKWV
SbAP2/1-249 2 ------------------------EPNNNQPA--ATAEEAAAGSGPGGEGGRKGKAPKGGPENGKFRYRGVRQRSWGKWV
consensus 81 . . . ....... . . ... . . .. .. ........ ................
 AP2 DOMAIN

AtABI4_wt 68 AEIREPRKRTRKWLGTFATAEDAARAYDRAAVYLYGSRAQLNLTPSSPSSVSSSSSSVSAASSPSTSSSSTQTLRPLLPR
**AtABI4 (soa1)** 68 AEIREPRKRTRKWLGTFATAEDAARAYDRAAVYLYGSRAQLNLTPSSPSSVSSSSSSVSAASSPSTSSSSTQTLRPLLPR

PtABI4/1-334 83 AEIREPRKRTRKWLGTFATAEDAARAYDRAAFILYGSRAHLNLQPS-----GSSSSAQSGSTSRNSTSSSSQTLRPLLPR
GmSGR/1-338 76 AEIREPRKRTRKWLGTFATAEDAARAYDRAAIILYGSRAQLNLQPS-----GSSSQ----SSSSRSSSSSTQTLRPLLPR
ZMAP2/1-248 55 AEIREPRKRSRKWLGTFATAEDAARAYDRAALLLYGPRAHLNLTSPPPPTLAAPRS--HPHSSAT--SSAPPALRPLLPR
SbAP2/1-249 56 AEIREPRKRSRKWLGTFATAEDAARAYDRAALLLYGPRAHLNLTSPPPPTLAAPRS--HPHSSATSSSSAPPALRPLLPR
consensus 161 .......................*.*....*. .*...* ... .. .... .. ................
 AP2 DOMAIN Ser/Thr (S/T) REPEATS

AtABI4_wt 148 PAAATVG------------------GGANFGP---YGI--PFNNNIFLNGGTSMLCPSYGFFPQQQQQQNQMVQMGQFQH
**AtABI4 (soa1)** 148 PAAATVG------------------GGANFGP---YGI—PFNNNIFLNGGTSMLCPSYGFFPQQQQQ-------------

PtABI4/1-334 158 PPGFGCGFGFTFSLSNPMASPSVTAASSGFTP---YGV-NCYSNNVV---GSALQCSSTNEMP-GQNHQQVMLQGYLIQH
GmSGR/1-338 147 PSGFTFNFPFNSAPATTVPYPYNNSHNYTYTPPVLYPIDNSKNHNIN---TVQVHHPHHYRCP-EEVVQVPHSESDLGGL
ZMAP2/1-248 131 PPLHQLS-----SDG--APAPDF-------------------------------------------HYHN-QFQ-RRLLP
SbAP2/1-249 134 PPLHHQL-----SSGACAPAPDF-------------------------------------------HHHHQQFQCHHLLP
consensus 241 . . . .... .. . ...... . . . . .. .
 Gln (Q) REPEATS

**AtABI4**_wt 205 QQYQNLHSNTNNNKISDIELTDVPVT----NSTSF---HHEVALGQE---QGGSGCNNNSSMEDLN-SLAGSVGSSLSI-
**AtABI4 (soa1)** 205 --------------------------------------------------------------------------------

PtABI4/1-334 230 ------GANTTNPNNIFVSSSVDPST----TTSYQ---NHCHRLPQH------------HAYDDVN-ALGGSVGSSFSLS
GmSGR/1-338 223 --------GGNNNIVVDGSIRSTSYQRHGFLDNINNNNNHVHVQVQHGVFSNQQQQHQNSVVEGVNSSVVGSVSSSMDA-
ZMAP2/1-248 159 ----------QPTPTLYYANTATAST----VTTSV---PTRVAVPQE---------------------------------
SbAP2/1-249 166 --------QAQPTPPLYYANTATAST----VTTTV---PTRVAVPQE---------------------------------
consensus 321 . . . ..... .. ... . . . ... . ... . ... .. ..

**AtABI4**_wt 273 -THPPPLVDPV-----CSMGLDPGYMVGDGSST-IWPFGG--EEEYSHNWGSIWDFIDPIL-GEFY--------------
**AtABI4 (soa1)** 273 --------------------------------------------------------------------------------
PtABI4/1-334 284 GSNTPPVVAPA-----GHLLQDPVMHIGPGSPS-AWN-----DEEYPP--PSIWDDEDPFL-FDF---------------
GmSGR/1-338 294 -SSVDPDLALV-------------GTMGLGSSSPFWSMAN--EDDYT---GSLWDYNDPFF-FDL---------------
ZMAP2/1-248 189 -----PAIAPAVGSSTSLQEPQVGTPEEARGEA-GWDYNGGEEEDYAA--ALLWDEPEPFFWFDVFLK------------
SbAP2/1-249 198 -----PAVAPA--------EPQASTPEEAPRET-GWDYHGGEEEDYAA--ALLWDEPEPFFWFDVFLK------------
consensus 401 .. . . . ..... .. .. .. . ...... ... .. . . ..............
 Pro (P) REPEATS ACIDIC DOMAIN
