# Supplementary material for: Arabidopsis suppressor mutant of abh1 shows a new face of the already known players: ABH1 (CBP80) and ABI4—in response to ABA and abiotic stresses during seed germination
Source: Plant Mol Biol. 2012 Nov 30;81(1):189–209. doi: 10.1007/s11103-012-9991-1 (PMC3527740; doi:10.1007/s11103-012-9991-1)

**Figure S6. Analysis of the number of stomata in original *abi4-101* mutant.**

1. The number of stomata


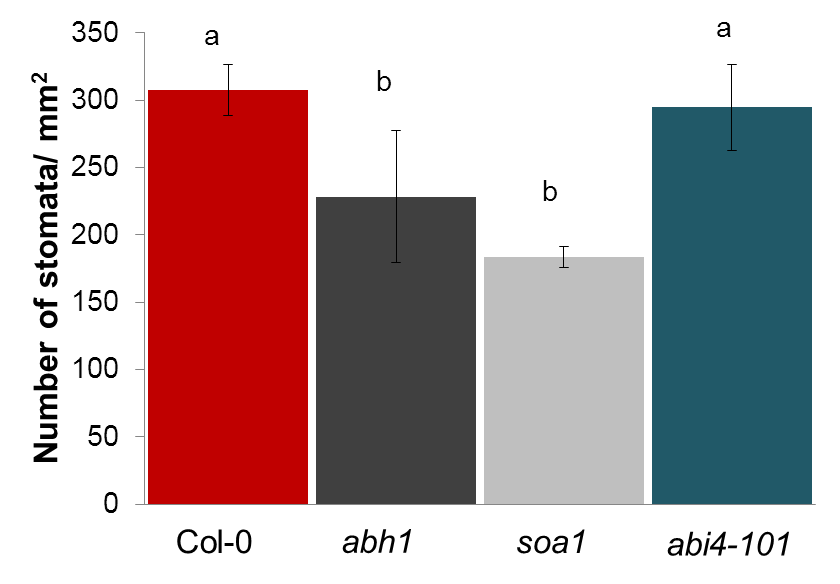


1. The number of stomata


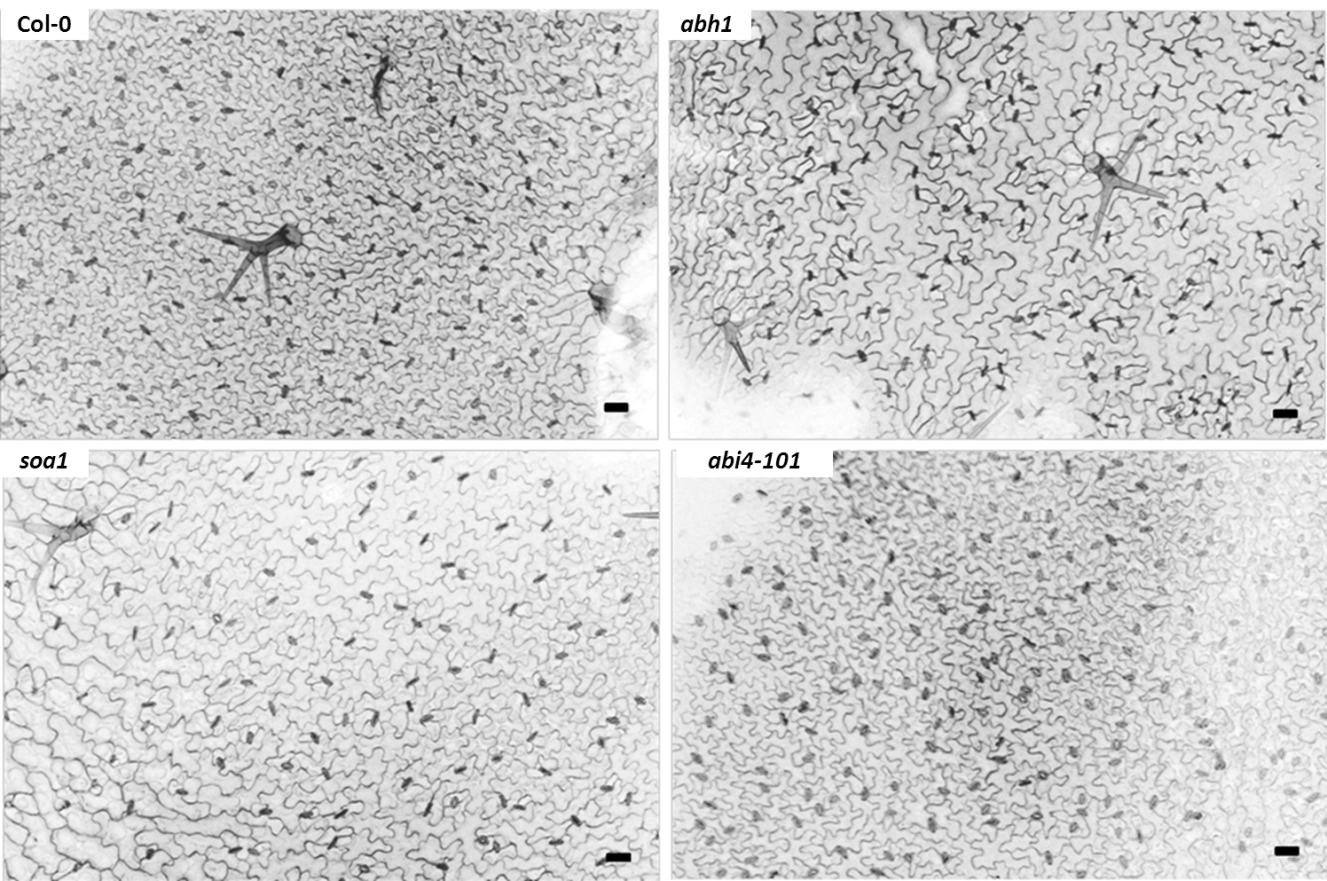

Supplement: Supplementary file 6 — Supplementary material 6 (DOC 1367 kb) [file 11103_2012_9991_MOESM6_ESM.doc]
