# Supplementary material for: Arabidopsis suppressor mutant of abh1 shows a new face of the already known players: ABH1 (CBP80) and ABI4—in response to ABA and abiotic stresses during seed germination
Source: Plant Mol Biol. 2012 Nov 30;81(1):189–209. doi: 10.1007/s11103-012-9991-1 (PMC3527740; doi:10.1007/s11103-012-9991-1)
Supplement: Supplementary file 7 — Supplementary material 7 (DOC 247 kb) [file 11103_2012_9991_MOESM7_ESM.doc]

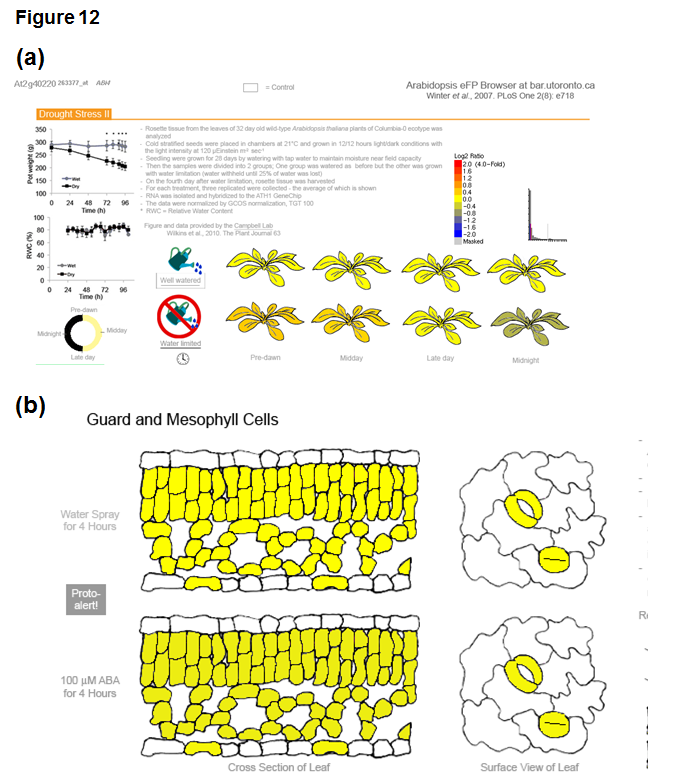
Figure S7. *In silico* analysis of the expression pattern of *ABI4* in drought stress (**a**) and guard cells (**b**) of *Arabidopsis thaliana* with the use of eFPBrowser.
