# Supplementary material for: Arabidopsis suppressor mutant of abh1 shows a new face of the already known players: ABH1 (CBP80) and ABI4—in response to ABA and abiotic stresses during seed germination
Source: Plant Mol Biol. 2012 Nov 30;81(1):189–209. doi: 10.1007/s11103-012-9991-1 (PMC3527740; doi:10.1007/s11103-012-9991-1)
Supplement: Supplementary file 8 — Supplementary material 8 (DOC 1535 kb) [file 11103_2012_9991_MOESM8_ESM.doc]

Figure S8. *In silico* analysis of expression pattern of *ABH1*, *ABI4*, *MYB33* and *MYB101* with the use of eFP Browser ([http://bar.utoronto.ca](http://bar.utoronto.ca/)).

1. *In silico* analysis of expression pattern of *ABH1*, *ABI4*, *MYB33* and *MYB101* during Arabidopsis development with the use of eFPBrowser.


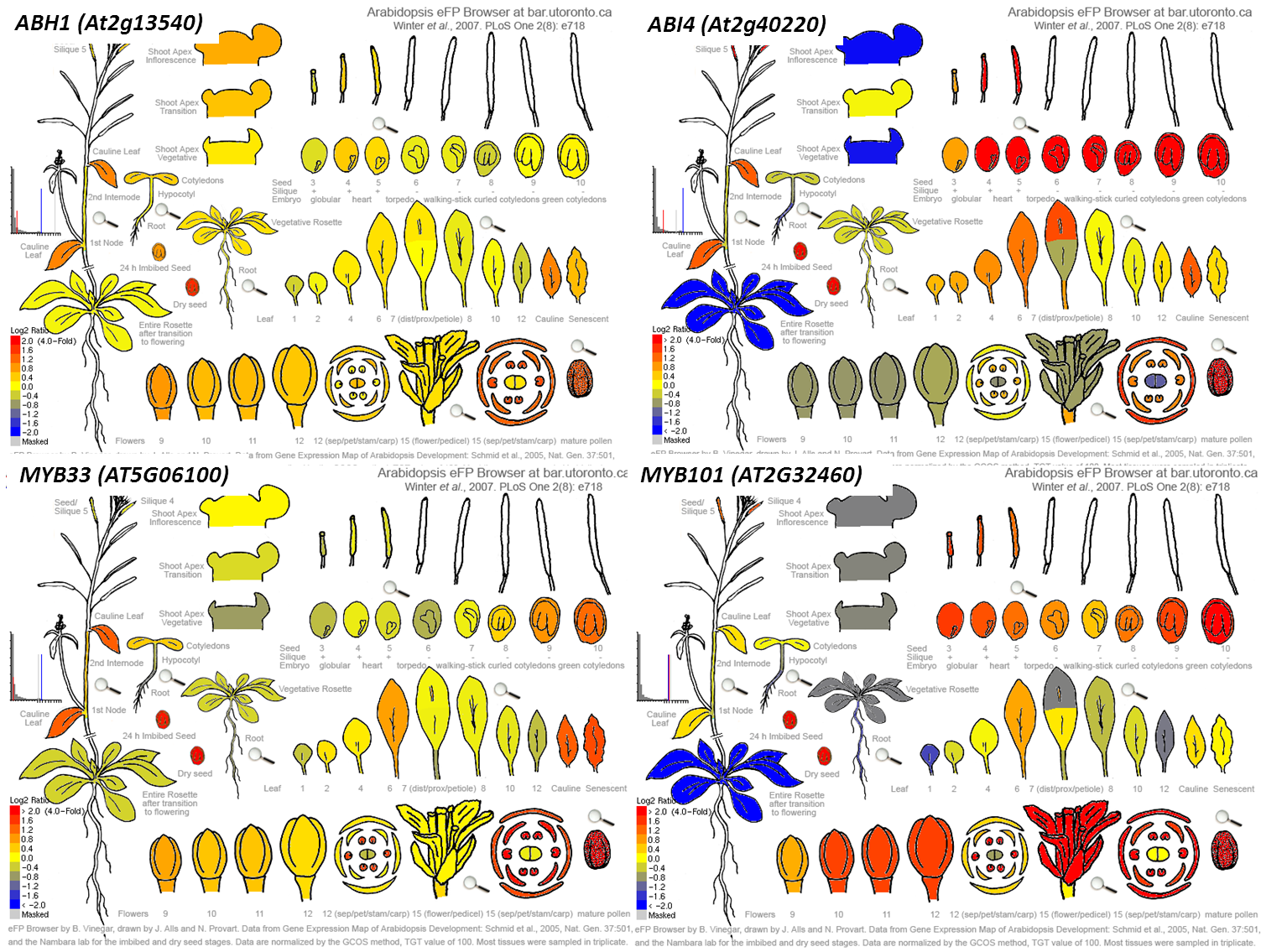


1. *In silico* analysis of expression pattern of *ABH1*, *ABI4*, *MYB33* and *MYB101* during seed imbibition.

**
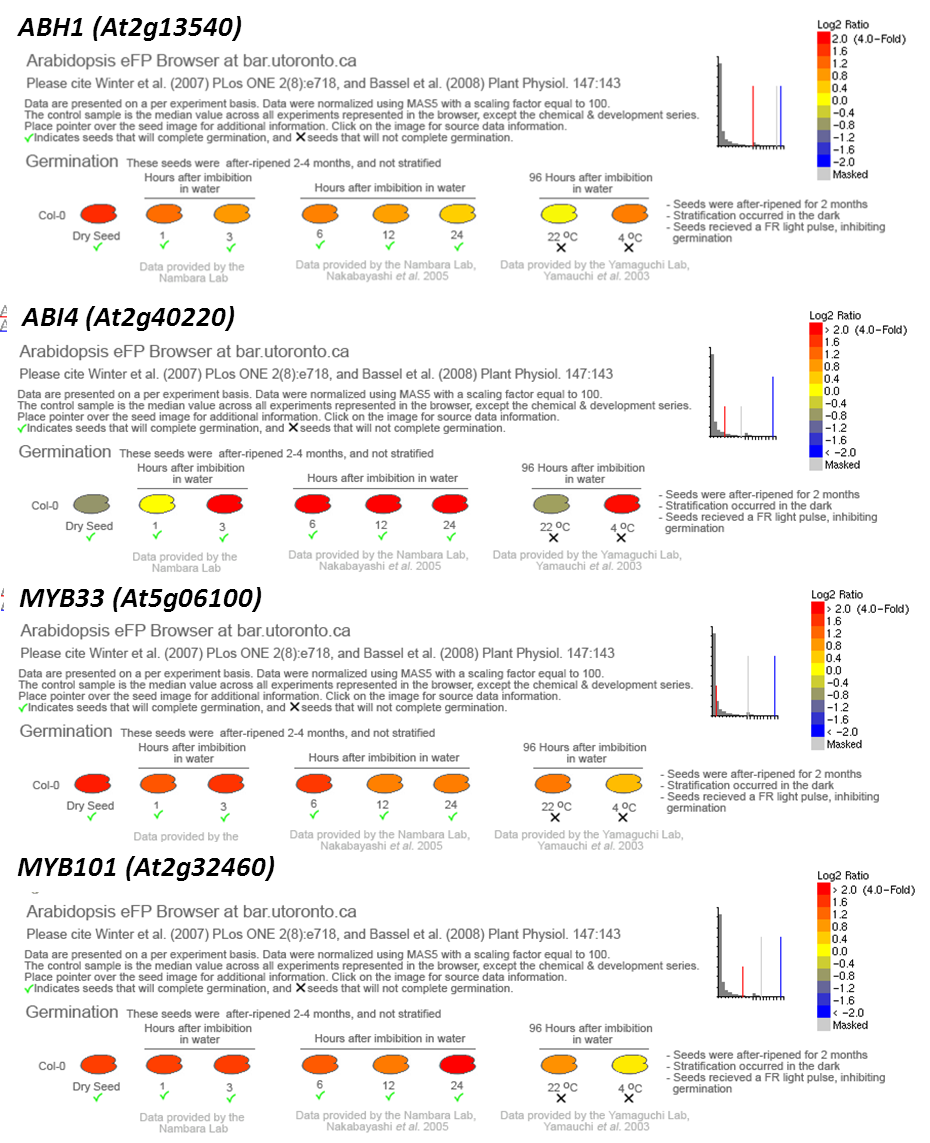
**
