# Supplementary material for: Arabidopsis suppressor mutant of abh1 shows a new face of the already known players: ABH1 (CBP80) and ABI4—in response to ABA and abiotic stresses during seed germination
Source: Plant Mol Biol. 2012 Nov 30;81(1):189–209. doi: 10.1007/s11103-012-9991-1 (PMC3527740; doi:10.1007/s11103-012-9991-1)

Figure S10. Phenotypic differences and similarities between *soa1* and its parental line *abh1*, wild-type Col-0 and *abi4-101* mutant. The bar is 1 mm.

1. Differences and similarities of flowers, stems and leaves of studied forms.


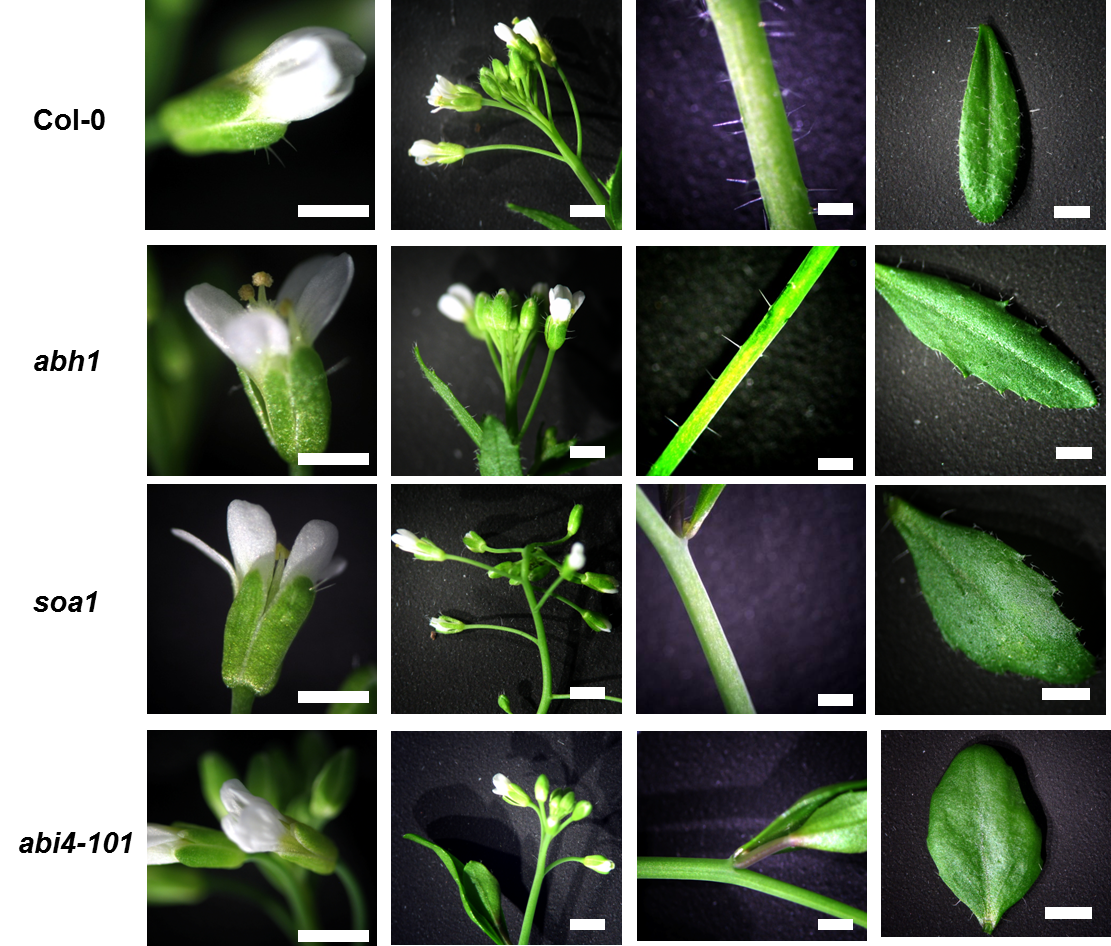


1. The phenotype of mature plants of studied genotypes.


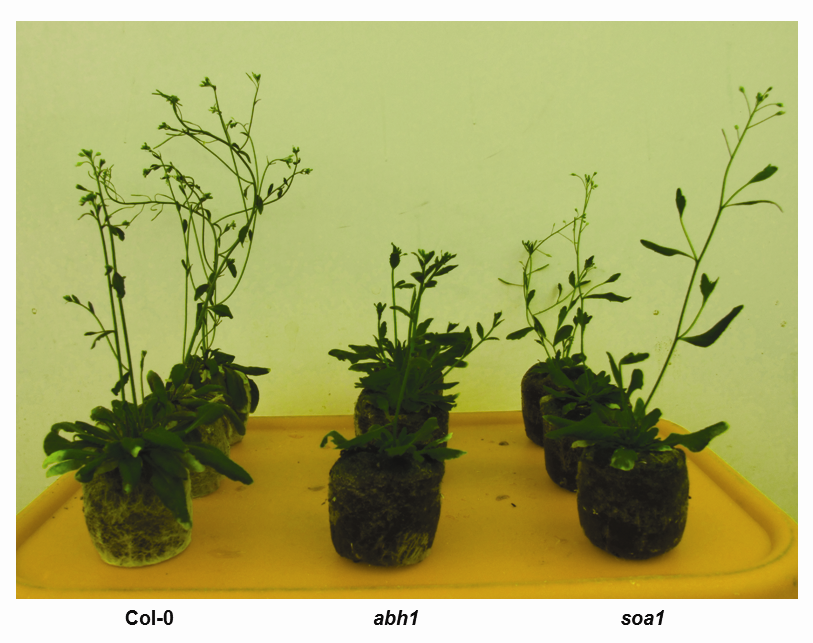

Supplement: Supplementary file 10 — Supplementary material 10 (DOC 2678 kb) [file 11103_2012_9991_MOESM10_ESM.doc]
