# Supplementary material for: Arabidopsis suppressor mutant of abh1 shows a new face of the already known players: ABH1 (CBP80) and ABI4—in response to ABA and abiotic stresses during seed germination
Source: Plant Mol Biol. 2012 Nov 30;81(1):189–209. doi: 10.1007/s11103-012-9991-1 (PMC3527740; doi:10.1007/s11103-012-9991-1)
Supplement: Supplementary file 11 — Supplementary material 11 (DOC 37 kb) [file 11103_2012_9991_MOESM11_ESM.doc]

**Table S1. Candidate genes chosen based on *soa1* phenotype.**

| **ID** | **Gene** | **AGI** | **Function** | **Chr#** | **Mutant** | **Phenotype** | **Reference** |
| --- | --- | --- | --- | --- | --- | --- | --- |
| 1. | *ABI1* | At4g26080 | ABI1 is involved in abscisic acid (ABA) signal transduction. It is a negative regulator of ABA promotion of stomatal closure. | 4 | *abi1-1* | Mutant displayed insensitivity to 10 µM ABA and reduced sensitivity to salt and osmotic stress during germination. | Koornneef et al. 1984; Leung et al. 1997 |
| 2. | *ABI2* | At5g57050 | ABI2 encodes a protein phosphatase 2C and is involved in ABA signal transduction. It binds fibrillin preprotein in vitro and in vivo. | 5 | *abi2-1* | Mutant displayed insensitivity to 10 µM ABA and reduced sensitivity to salt and osmotic stress during germination. | Koornneef et al. 1984; Leung et al. 1997 |
| 3. | *ABI3* | At3g24650 | ABI3 is a central regulator in ABA signaling essential for seed maturation as seed-specific transcriptional activator. It is a regulator of the transition between embryo maturation and early seedling development. | 3 | *abi3-4* | Mutant displayed reduced sensitivity to ABA and normal plant growth. | Giraudat et al. 1992 |
|  |  |  |  |  | *abi3-5* | Mutant displayed reduced sensitivity to ABA and normal plant growth. | Ooms et al. 1993 |
|  |  |  |  |  | *abi3-1* | Mutant displayed insensitivity to 10 µM ABA and reduced sensitivity to salt and osmotic stress during germination. | Koornneef et al. 1984 |
| 4. | *ABI4* | At2g40220 | *ABI4 e*ncodes a member of the DREB subfamily A-3 of ERF/AP2 transcription factor family. It is involved in ABA signal transduction and ABA-mediated glucose response. | 2 | *abi4-1* | Mutant displayed insensitivity to 5 µM ABA and reduced sensitivity to salt and osmotic stress during germination. Also it is not wilty when exposed to drought. | Finkelstein, 1994 |
|  |  |  |  |  | Other *abi4* mutants described in **TABLE S3** | | |
| 5. | *ABI5* | At2g36270 | *ABI5* encodes a member of the basic leucine zipper transcription factor family. It is involved in ABA signalling during seed maturation and germination. | 2 | *abi5-1* | Mutant displayed abscisic acid insensitivity to 3 µM ABA and reduced sensitivity to salt and osmotic stress during germination. Also it is not wilty when exposed to drought. | Finkelstein and Lynch, 2000 |
| 6. | *CHlH* | At5g13630 | *CHlH* encodes magnesium chelatase involved in plastid-to-nucleus signal transduction. | 5 | *cch1-1* | Mutant showed weak ABA insensitivity during seed germination and growth after germination. | Xu et al. 2011 |
