# Supplementary material for: Arabidopsis suppressor mutant of abh1 shows a new face of the already known players: ABH1 (CBP80) and ABI4—in response to ABA and abiotic stresses during seed germination
Source: Plant Mol Biol. 2012 Nov 30;81(1):189–209. doi: 10.1007/s11103-012-9991-1 (PMC3527740; doi:10.1007/s11103-012-9991-1)
Supplement: Supplementary file 12 — Supplementary material 12 (DOC 40 kb) [file 11103_2012_9991_MOESM12_ESM.doc]

| Table S2. *ABI4* identified alleles with the use of different approaches based on literature data. | | | | | |
| --- | --- | --- | --- | --- | --- |
| **The investigated trait/ metabolic pathway** | **Mutagenesis** | **Ecotype** | **Type of selection/ stage of development** | **Identified alleles/ mutation effect** | **References** |
| **ABA insensitivity** | γ irradiation | Col-0 | Seed germination at the presence of 3 μM ABA | *abi4-1/* frame shift (G463Δ) leading to truncated protein | Finkelstein, 1994; Finkelstein et al. 1998 |
| **Insensitivity to salt** | fast neutrons | Col-0 | Seed germination at the presence of:  NaCl (0–250 mM), KCl (0–150 mM), Na2SO4 (0–150 mM), mannitol (0–500 mM), or ABA (2.5–50 μM). | *sãn5* (*abi4-2*)/ frame shift (A277Δ) leading to truncated protein | Quesada et al. 2000 |
| **Insensitivity to sucrose** | Chemical mutagenesis with EMS of transgenic lines PC::LUC**^A^** | C24 | Increased activity of luciferase during seed germination at the presence of 3% sucrose; seed germination at the presence of 5 mM mannose; 6% glucose | *sun6-1* (*abi4*)/ (G237A) premature stop codon leading to truncated protein | Huijser et al. 2001 |
| **Insensitivity to sucrose** | Chemical mutagenesis with EMS of transgenic lines ApL3::P450**^B^** | Col-0 | Seed germination and seedlings development at the presence of 100 mM sucrose and R7402 | *isi3-1* (*abi4*)/ (C562T) premature stop codon leading to truncated protein  *isi3-2* (*abi4-104*)/ (G205A) E69K | Rook et al. 2001 |
| **Insensitivity to glucose** | Insertional mutagenesis | Col-0 | Seed germination and seedlings development at the presence of 7% glucose | *gin6* (*abi4*)/ knock-out | Arenas-Huertero et al. 2000 |
| **Insensitivity to sugars** | Chemical mutagenesis with EMS | Col-0 | Seed germination and seedlings development at the presence of 0,3 M sucrose i 0,3 M glucose | *sis5-1* (*abi4-101*)/ (C577T) premature stop codon leading to truncated protein  *sis5-2* (*abi4-102*)/ (C240T) premature stop codon leading to truncated protein  *sis5-3* (*abi4-103*)/ (C115T) premature stop codon leading to truncated protein  *sis5-4* (*abi4-104*)/ (G205A) E69K | Laby et al. 2000;  Gibson et al. 2001 |

**^A^** It was showed that seedlings germinated from seeds incubated in darkness displayed inreased level of *PC* (Plastocyanin) and other photosynthesis involved genes expression. An addition of sucrose to the medium results in repression of their expression (Dijkwel i in., 1996). Huijser and co-workers (2001) performed EMS mutagenesis of transgenic lines PC::LUC and screened transformants for increased LUC activity at the presence of 3% sucrose. **^B^** Sucrose induces *ApL3* gene expression, involved in starch biosynthesis. Rook and co-workers (2001) performed EMS mutagenesis of transgenic lines ApL3::P450. Bacterial cytochrome (P450), used as a marker gene, performed non-toxic R7402 into toxic causing letality. Seeds of transformants were screened at the presence of sucrose and R7402.
