# Supplementary material for: Arabidopsis suppressor mutant of abh1 shows a new face of the already known players: ABH1 (CBP80) and ABI4—in response to ABA and abiotic stresses during seed germination
Source: Plant Mol Biol. 2012 Nov 30;81(1):189–209. doi: 10.1007/s11103-012-9991-1 (PMC3527740; doi:10.1007/s11103-012-9991-1)
Supplement: Supplementary file 13 — Supplementary material 13 (DOC 28 kb) [file 11103_2012_9991_MOESM13_ESM.doc]

Table S3. Analysis of the co-segregation of leaf serration with *abh1* hypersensitivity to ABA in F_2_ progeny ***abh1* x Col-0**.

| **Number of F_2_ plants** | | | |
| --- | --- | --- | --- |
| **Total analyzed** | **Hypersensitive to ABA/ serrated leaves** | **Insensitive to ABA/ rounded leaves** | **χ^2^ _3:1_** |
| **120** | 29 | 91 | 0,04^*^ |

*P=0,05
