# Supplementary material for: Arabidopsis suppressor mutant of abh1 shows a new face of the already known players: ABH1 (CBP80) and ABI4—in response to ABA and abiotic stresses during seed germination
Source: Plant Mol Biol. 2012 Nov 30;81(1):189–209. doi: 10.1007/s11103-012-9991-1 (PMC3527740; doi:10.1007/s11103-012-9991-1)
Supplement: Supplementary file 14 — Supplementary material 14 (DOC 51 kb) [file 11103_2012_9991_MOESM14_ESM.doc]

Table S4. Primer sequences used in the experiments.

| **Gene** | **FORWARD PRIMER** | **REVERSE PRIMER** |
| --- | --- | --- |
| ***ABI1*** | GAACCATTGAGAAGCTTTATGGA | GATTGGATCTTTTGATTAGGGTTG |
| ***ABI2*** | TCGGAAACTGCAAAAACTTG | TCATGGATCATTATCAAGAGAAAGT |
| ***ABI3*** | TGGAGTAAACCCAAACGGT | TCAGGAAGCGTTGGGAGA |
|  | TTGTTTCATT TCCACTTCAACG | CATTTGCATGTCTCCACCAC |
|  | CGAGTTCTTTGACACCTCAGC | CGGATTCA TGTTGTATCCATTG |
|  | CACCACCACAGTCTGGTCC | CCGGTGT TCTCGAGGAGATA |
|  | GCAGAAAGTCTTGAAGCAAAGC | CCGACTCGACAAGAAAAAGC |
| ***ABI4*** | GGACCCTTTAGCTTCCCAAC | TGATACTGTTGGTGTTGGAATTG |
|  | CGTAGGAG GAGGAGCCAACT | AATTCCCCCAAGATGGGAT |
| ***ABI5*** | GGTGGTTCTAGAGGCAACGA | TCCTCCTCTGTCTTCTCTCCA |
| ***CHlH*** | TTGTTGGTTGGTCTCTCA | TGAACTACAAAGAAAATTCACTC |
| ***pre-miR159a*** | TCTTTACAGTTTGCTTATGTCAGATCCA | ACCCTGCTCAACTCATGTTTGAA |
| ***pre-miR159b*** | TGCTTGGATCTCTAATGCTGTTCA | TCACCCTGCTAAACCCTCCA |
| ***pre-miR159c*** | ACGAAGAGGACAAGATTTGAGGAA | AGGTCGTGCAATCCCCTTAAAGT |
| ***MYB33*** | TTCCTTATTTAGGGTTTGTTTTCC | GGCCTTTCTTGAGAGCATGA |
| ***MYB101*** | CAAGAACTATTGGAACACGAGG | TTAGTGTACTGAATCATATTTTGGTGG |
| ***TSPO*** | TGTGTATGCATCAGACATGAGC | CGTTTCGGCGACATAAAAGT |
| ***PYL2F1*** | CTTTCATAAAACCCTCACACG | TGATTTGCCGTTTATTGGAA |
| ***F3G5*** | CATTTGCCCCATAAATACCAA | TTAACCGAAGCAACCAAACC |
|  | GTGAATTTGTAGGAGTTGGATGG | GTGAATTTGTAGGAGTTGGATGG |
| ***PYL4*** | CACCACTCACATGAACTCTGC | AACAACGCTGAAGCTGATGA |
|  | CGATATCCTCGACGACGAAC | GGCCAACAACTCAATGTAACG |
| ***EM6*** | GAACATCCACGATGCAACAC | TGCGTACTTTTCTTTGGATGC |
| ***LOS2*** | GGTTTTGGCTAAATCCGTGA | ACACAGCAAGAATCGCATTG |
|  | ATGAACTTGACGGAACCCAA | CAAAGAGCGCAACAGCTACA |
|  | CAGCTTGTCAGGATAGAGTGTGA | AGTCATGAACACAAGCAGGTCT |
| ***ABI3 qRT*** | AGCAAAGCAGTGGGTAAC | AACCTGTAGCGCATGTTCCAAAC |
| ***ABI4 qRT*** | ACTTCCTCCGCTCAACGCAAAC | AACGCCACGGTAACGGAACTTG |
| ***ABI5 qRT*** | AAACATGCATTGGCGGAGTTGG | CGGTTGTGCCCTTGACTTCAAAC |
| ***RAB18*** | TCGGTCGTTGTATTGTGCTTTTT | CCAGATGCTCATTACACACTCATG |
| ***RD29B*** | GGCGGGCAAAGCGAG | TGCCCGTAAGCAGTAACAGATC |
